# The Fab Conformations in the Solution Structure of Human Immunoglobulin G4 (IgG4) Restrict Access to Its Fc Region: IMPLICATIONS FOR FUNCTIONAL ACTIVITY*[Fn FN2]

**DOI:** 10.1074/jbc.M114.572404

**Published:** 2014-05-29

**Authors:** Lucy E. Rayner, Gar Kay Hui, Jayesh Gor, Richard K. Heenan, Paul A. Dalby, Stephen J. Perkins

**Affiliations:** From the ‡ Department of Structural and Molecular Biology, Division of Biosciences, Darwin Building and; ¶ Department of Biochemical Engineering, Division of Engineering, Roberts Building, University College London, Gower Street, London WC1E 6BT, United Kingdom and; § ISIS Facility, Science and Technology Facilities Council, Rutherford Appleton Laboratory, Harwell Oxford, Didcot OX11 0QX, United Kingdom

**Keywords:** Analytical Ultracentrifugation, Antibody, Complement, Neutron Scattering, Protein Structure, X-ray Scattering, Constrained Modeling, Human IgG4

## Abstract

Human IgG4 antibody shows therapeutically useful properties compared with the IgG1, IgG2, and IgG3 subclasses. Thus IgG4 does not activate complement and shows conformational variability. These properties are attributable to its hinge region, which is the shortest of the four IgG subclasses. Using high throughput scattering methods, we studied the solution structure of wild-type IgG4(Ser^222^) and a hinge mutant IgG4(Pro^222^) in different buffers and temperatures where the proline substitution suppresses the formation of half-antibody. Analytical ultracentrifugation showed that both IgG4 forms were principally monomeric with sedimentation coefficients *s*_20,*w*_^0^ of 6.6–6.8 S. A monomer-dimer equilibrium was observed in heavy water buffer at low temperature. Scattering showed that the x-ray radius of gyration *R_g_* was unchanged with concentration in 50–250 mm NaCl buffers, whereas the neutron *R_g_* values showed a concentration-dependent increase as the temperature decreased in heavy water buffers. The distance distribution curves (*P*(*r*)) revealed two peaks, M1 and M2, that shifted below 2 mg/ml to indicate concentration-dependent IgG4 structures in addition to IgG4 dimer formation at high concentration in heavy water. Constrained x-ray and neutron scattering modeling revealed asymmetric solution structures for IgG4(Ser^222^) with extended hinge structures. The IgG4(Pro^222^) structure was similar. Both IgG4 structures showed that their Fab regions were positioned close enough to the Fc region to restrict C1q binding. Our new molecular models for IgG4 explain its inability to activate complement and clarify aspects of its stability and function for therapeutic applications.

## Teaser

**Background:** The human IgG4 antibody subclass does not activate complement and forms half-antibodies.

**Results:** Ultracentrifugation and x-ray/neutron scattering together with atomistic modeling revealed asymmetric concentration-dependent IgG4 solution structures.

**Conclusion:** The complement and receptor Fc binding sites are hindered by the Fab regions, explaining loss of activity.

**Significance:** These solution structures clarify IgG4 function and its therapeutic applications.

## Introduction

IgG4 is the only human IgG antibody subclass that does not activate complement (1) and is able to undergo Fab-arm exchange (2) and associate with other IgG molecules via its Fc region (3). Interest in IgG4 arises from its distinct properties compared with the other human IgG subclasses IgG1, IgG2, and IgG3 (4) and its desirability as a pharmaceutical (5). Although the specific function of each IgG subclass remains to be defined, different antigens (*e.g.* protein, polysaccharide, and allergen) activate varied responses from the four IgG subclasses. IgG4 plasma levels rise after prolonged antigen exposure, leading to anti-inflammatory effects (6). Fab-arm exchange causes IgG4 to behave as if it were monovalent (7), in turn preventing the cross-linking of antibody-antigen complexes and explaining the anti-inflammatory effects of IgG4. IgG4 is popular as a therapeutic as it does not activate complement. However, IgG4 drug products can perform Fab-arm exchange with endogenous IgG4, limiting their efficacy (8). IgG4 can bind to each of the Fc receptor subclasses; however, it does so weakly in comparison with other subclasses (9).

IgG4 Fab-arm exchange is mediated through the Fc region (hinge, C_H_2, and C_H_3 domains) (10) in which the separation of the two heavy chains to form half-antibodies (half-mers) is attributed to Ser^222^ in its core hinge region (supplemental Fig. S1). Cys^220^ and Cys^223^ in the core hinge form disulfide bridges between the heavy chains. Mutation of Ser^222^ to Pro^222^ by genetic engineering gives an IgG1-like core hinge and eliminates half-mer formation (11). Conformational modeling on the two core hinge peptides predicted that the wild-type IgG4(Ser^222^) hinge is more flexible than the mutant IgG4(Pro^222^) hinge, perhaps explaining the formation of stable intrachain disulfide bonds in the mutant (12). The equilibrium between these disulfide bridges leads to heterogeneous IgG4 populations, each with two different heavy chains (13). The kinetic rates of half-mer formation *in vitro* are very low, and Fab-arm exchange does not readily occur. This slow exchange is attributed to strong non-covalent contacts between the C_H_3 domains. In fact Fab-arm switching is observed predominantly *in vivo*.

Fab-arm exchange has been clarified by mutagenesis studies that showed Arg^409^ to be crucial for stabilizing the C_H_3-C_H_3 interaction (Arg^403^ in supplemental Fig. S2G) (14–16). Fab-arm switching might be facilitated by protein-disulfide isomerase or binding to the Fc receptor FcRn^2^ (4). It has now been shown that Fab-arm exchange requires a combination of factors, predominantly the C_H_3 domains and the hinge region, and the presence of a reducing environment; adjuvants are not necessary. IgG1 undergoes Fab-arm switching after replacement of the IgG1 C_H_3 domain by the IgG4 C_H_3 domain, showing that the IgG4 C_H_3 domain is crucial for exchange (17). Interestingly, the Fab regions stabilize the intact IgG4 structure (18). The ability of IgG4 to self-associate is also of interest. IgG4-Fc can interact with IgG1-Fc when IgG1 is coupled to a solid support; the coupling of IgG1 is thought to induce a conformational change, allowing accessibility of its IgG1-Fc for IgG4 binding (3).

An understanding of IgG4 would be facilitated by its atomistic solution structure. Atomistic antibody structures are determined using our joint strategy of x-ray and neutron scattering coupled with analytical ultracentrifugation and constrained modeling based on crystal structures (19–21). Joint x-ray and neutron scattering allows the study of a broad range of different solution conditions, and this is facilitated by the ability to perform x-ray measurements in seconds rather than minutes. Previously IgG4(Ser^222^) had been studied by ultracentrifugation and x-ray scattering to show that IgG4 undergoes a concentration-dependent Fab-arm rearrangement (20). This concentration dependence is explored here using more detailed solution structural data for both IgG4(Ser^222^) and IgG4(Pro^222^). We show that IgG4(Ser^222^) and IgG4(Pro^222^) are principally monomeric, but IgG4(Ser^222^) shows self-associative behavior in heavy water. Both forms display concentration-dependent structural changes corresponding to Fab-arm rearrangements at low concentrations. Both the IgG4(Ser^222^) and IgG4(Pro^222^) solution structures display extended asymmetric arrangements of the Fab regions relative to the Fc region. Our three independently determined IgG4 solution structures showed that the Fab regions were close enough to the Fc region to hinder the C1q and FcR binding sites at the top of the Fc region in IgG4. The implications of this result for the reduced immune reactivity of IgG4 are discussed.

## EXPERIMENTAL PROCEDURES

### 

#### 

##### Purification and Composition of IgG4

A wild-type human-mouse chimera, IgG4 B72.3 with Ser^222^, was generously supplied by Dr. Jim Davies (Lonza Biologics) (supplemental Figs. S1 and S2). Other studies named Ser^222^ as Ser^228^ (22), Ser^229^ (12), and Ser^241^ (23). A mutant IgG4 with Pro^222^ was generously supplied by Dr. Bryan Smith (UCB) (24). Both IgG4(Ser^222^) and IgG4(Pro^222^) were further purified by gel filtration to remove nonspecific aggregates using a Superose 6 10/300 column (GE Healthcare), concentrated using an Amicon Ultra spin concentrator (50-kDa-molecular mass cutoff), and dialyzed at 4 °C against the measurement buffer (see below). The sequence identity between both IgG4 molecules was 100% for the C_H_1, C_H_2, and C_H_3 domains (except for the S222P substitution). Different identities were found for the V_H_ (67.5%), V_L_ (59.3%), and C_L_ (96.2%) domains, giving an overall identity between the two IgG4 sequences of 86.7% (supplemental Fig. S2). The *N*-linked oligosaccharides at Asn^291^ on the C_H_2 domains (supplemental Fig. S2) were assumed to be a complex-type biantennary oligosaccharide structure with a Man_3_-GlcNAc_2_ core and two NeuNAc-Gal-GlcNAc antennae (25, 26). The IgG4(Ser^222^) molecular mass was calculated to be 147.0 kDa, its unhydrated volume was 188.7 nm^3^, its hydrated volume was 248.7 nm^3^ (based on a hydration of 0.3 g of water/g of glycoprotein and an electrostricted volume of 0.0245 nm^3^ per bound water molecule), its partial specific volume *v* was 0.728 ml/g, and its absorption coefficient was 14.5 (1%, 1-cm pathlength, 280 nm). For IgG4(Pro^222^), the molecular mass was 149.5 kDa, *v* was 0.728 ml/g, and the absorption coefficient was 14.1 (27).

All data were recorded in phosphate-buffered saline with different NaCl concentrations. That termed PBS-137 comprised 137 mm NaCl, 8.1 mm Na_2_HPO_4_, 2.7 mm KCl, and 1.5 mm KH_2_PO_4_ (pH 7.4). Buffers containing 50 mm NaCl or 250 mm NaCl in place of 137 mm NaCl were termed PBS-50 and PBS-250, respectively. The buffer densities were measured using an Anton Paar DMA 5000 density meter for comparison with the theoretical values from SEDNTERP (28). Measured densities were 1.00544 g/ml for PBS-137 at 20 °C (theoretical, 1.00534 g/ml), 1.00176 g/ml for PBS-50 at 20 °C (theoretical, 1.00175 g/ml), and 1.01029 g/ml for PBS-250 at 20 °C (theoretical, 1.00998 g/ml). For PBS-137 in heavy water, recorded values were 1.113137 g/ml at 6 °C, 1.11238 g/ml at 20 °C, and 1.109986 g/ml at 30 °C.

##### Sedimentation Velocity Data for IgG4

Analytical ultracentrifugation data for both IgG4 forms were obtained on two Beckman XL-I instruments equipped with AnTi50 rotors. Sedimentation velocity data were acquired for IgG4(Ser^222^) samples in PBS-50, PBS-137, and PBS-250 at 20 °C; in PBS-137 at 6, 20, and 30 °C; and in PBS-137 with 100% ^2^H_2_O. Data were collected at rotor speeds of 40,000 and 50,000 rpm in two-sector cells with column heights of 12 mm. Sedimentation velocity data were acquired for IgG4(Pro^222^) in PBS-137 at 20 °C only. Data were collected at rotor speeds of 30,000, 40,000, 50,000, and 60,000 rpm. Sedimentation analysis was performed using direct boundary Lamm fits of up to 700 scans using SEDFIT (version 12.1) (29, 30). SEDFIT resulted in size distribution analyses *c*(*s*) that assume all species to have the same frictional ratio *f*/*f*_0_. The final SEDFIT analyses used a fixed resolution of 200 and optimized the *c*(*s*) fit by floating *f*/*f*_0_ and the baseline until the overall root mean square deviation and visual appearance of the fits were satisfactory. The percentage of oligomers in the total loading concentration was derived using the *c*(*s*) integration function.

##### X-ray and Neutron Scattering Data for IgG4

X-ray scattering data were obtained during a single beam session in 16-bunch mode on Instrument ID02 at the European Synchrotron Radiation Facility, Grenoble, France, operating with a ring energy of 6.0 GeV (31). Data were acquired using a fiber optically coupled high sensitivity and dynamic range charge-coupled device detector (FreLoN) with a resolution of 512 × 512 pixels. A sample-to-detector distance of 3.0 m was used. IgG4(Ser^222^) in PBS-50, PBS-137, and PBS-250 was studied at 20 °C at eight concentrations between 0.48 and 6.91 mg/ml (3.3–47 μm) in which 100-μl samples were measured in a polycarboxylate capillary (2-mm diameter) that avoids protein deposits during beam exposures. The sample was moved continuously during beam exposure to eliminate radiation damage. Sets of 10 time frames, each of duration 0.1 or 0.2 s/frame, were acquired in quadruplicate as a control of reproducibility. Online checks during data acquisition confirmed the absence of radiation damage after which the 10 frames were averaged. In the same beam session, IgG4(Pro^222^) in PBS-137 at 20 °C was studied at concentrations between 0.5 and 2.0 mg/ml (3.3-13.4 μm).

Neutron scattering data were obtained on Instrument SANS2D at the ISIS pulsed neutron source at the Rutherford Appleton Laboratory, Didcot, UK (32). A pulsed neutron beam was derived from proton currents of ∼40 μA. SANS2D scattering and transmission data were recorded with a collimation of 4 m, a sample-to-detector distance of 4 m, a beam diameter of 12 mm, and a wavelength range of 0.175–1.65 nm that was determined by time of flight. IgG4(Ser^222^) samples were measured in 2-mm-path length circular banjo cells in a thermostated rack at 20 °C as well as at 6 and 37 °C. Data acquisitions lasted ∼1–2 h for four samples at concentrations between 2.0 (14 μm) and 8.0 mg/ml (55 μm) in PBS-137 prepared in 100% heavy water.

In a given solute-solvent contrast, the radius of gyration *R_g_* is a measure of structural elongation if the internal inhomogeneity of scattering densities within the protein has no effect. Guinier analyses at low *Q* (where *Q* = 4π sin θ/λ; 2θ is the scattering angle, and λ is the wavelength) gives the *R_g_* and the forward scattering at zero angle *I*(0) (33).

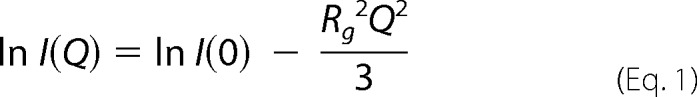
 This expression is valid in a *Q*·*R_g_* range up to 1.5. If the structure is elongated, the mean radius of gyration of cross-sectional structure *R*_XS_ and the mean cross-sectional intensity [*I*(*Q*)*Q*]*_Q_*_→0_ is obtained from Equation 2.

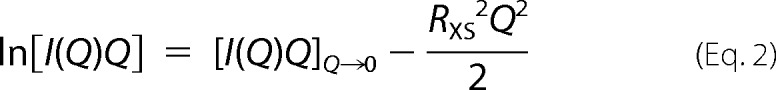
 For immunoglobulins, the cross-sectional plot exhibits two regions, a steeper innermost region and a flatter outermost region (34), identified by *R*_XS-1_ and *R*_XS-2_, respectively. The *R_g_* and *R*_XS_ analyses were performed using an interactive PERL script program SCTPL7^3^ on Silicon Graphics OCTANE Workstations. Indirect transformation of the scattering data *I*(*Q*) in reciprocal space into real space to give the distance distribution function *P*(*r*) was carried out using the program GNOM (35).

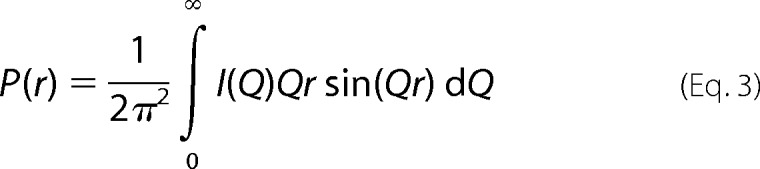

*P*(*r*) corresponds to the distribution of distances *r* between all volume elements. This yields the maximum dimension of the macromolecule *L* and its most commonly occurring distance vector *M* in real space. To calculate this, the x-ray *I*(*Q*) curve utilized up to 341 data points in the *Q* range between 0.03 and 1.60 nm^−1^. The neutron *I*(*Q*) curve utilized up to 112 data points in the *Q* range between 0.01 and 2.0 nm^−1^.

##### Debye Scattering and Sedimentation Coefficient Modeling of IgG4

A total of 20,000 conformationally randomized full sized IgG4(Ser^222^) models were created by joining molecular structures for the IgG4 Fab and Fc regions. A homology model for human IgG4 was constructed from crystal structures for Fab B72.3 and human IgG1 (Protein Data Bank codes 1BBJ and 1HZH) (36, 37) as described previously (20). Four sets of 5000 randomized IgG4 models were created using different hinge lengths and compositions. Two sets of asymmetric models were based on the IgG1 crystal structure. For these, a 9-residue hinge peptide, ^212^VESKYGPPC^220^, was constrained to be of a minimum length between 2.2 and 3.1 nm or 3.1 and 3.15 nm where the latter is almost fully extended in length. As Cys^220^ is located asymmetrically relative to the Fc structure, all these models were conformationally asymmetric. Because this asymmetry may be a crystallographic artifact, two sets of symmetric models were created. For these, a 21-residue hinge peptide, ^212^VESKYGPPCPSCPAPEFLGGP^232^, that encompassed the whole hinge was constrained to be of a minimum length between 5.0 and 7.0 nm or between 7.0 and 7.35 nm where the latter is almost fully extended in length. Because Pro^232^ is located symmetrically in relation to the Fc structure, the resulting IgG4 models contained Fab regions in both symmetric and asymmetric orientations. The outermost hinge residues were anchor points used to superimpose each hinge conformation onto the Fab and Fc regions to create the full-length IgG4 model. For each of the four sets of 5000 IgG4 models, two different hinge conformations were randomly selected to join the two Fab and Fc regions to form IgG4.

The scattering curve was calculated from each IgG4 model using sphere models. A cube side of 0.557 nm and a cutoff of four non-hydrogen atoms was used to convert the atomic coordinates into 1142 spheres that corresponded to the unhydrated structure seen by neutron scattering. Because hydration shells are visible by x-rays, a hydration shell corresponding to 0.3 g of water/g of protein was created using HYDRO (38), giving an optimal total of 1468 spheres. The x-ray scattering curve *I*(*Q*) was calculated using the Debye equation adapted to spheres (39). Details are given elsewhere (26, 40). Steric overlap between the Fab and Fc regions was assessed using the number of spheres *n* in each model where models showing less than 95% of the required total of 1468 spheres (x-ray) or 1142 spheres (neutrons) were discarded. Of the 20,000 hydrated models, 82% showed no steric overlap. The x-ray *R_g_*, *R*_XS-1_, and *R*_XS-2_ values were calculated from the modeled curves in the same *Q* ranges used for the experimental Guinier fits. Models that passed *R_g_* and *R*_XS_ filters of ±5% of the experimental value were then ranked using a goodness-of-fit *R*-factor (defined by analogy with protein crystallography) in the *Q* range extending to 1.6 nm^−1^. For the neutron modeling, 84% of the 20,000 unhydrated sphere models showed no steric overlap. The neutron models were assessed as for the x-ray scattering models above with corrections for wavelength spread and beam divergence, but no correction was required for a flat background caused from incoherent scattering because of the extrapolation to zero concentration.

Sedimentation coefficients (*s*_20,*w*_^0^) were calculated directly from the hydrated Debye sphere models using the program HYDRO (41). They were also calculated from the atomic coordinates in the HYDROPRO shell modeling program using the default value of 0.31 nm for the atomic element radius for all atoms to represent the hydration shell (42). Previous applications of these calculations to antibodies are reviewed elsewhere (43).

The outcome of the fit searches was evaluated by calculating the distances between the centers of mass of the Fab-Fc and Fab-Fab pairs (d1, d2, and d3; excluding hydrogen atoms) using a Python script. The three angles between the Fab and Fc regions were also calculated; these were defined in a Python script as the angle of intersection from the dot product between two vectors. Each vector was defined as the long axis through each Fab or Fc region, each defined as the line passing through the centers of gravity between each cluster of four cysteine α-carbon atoms at the two ends of the Fab and Fc regions. One cluster at each end of each Fab or Fc region corresponded to the conserved disulfide bridge in each immunoglobulin fold domain. Artwork was prepared using PyMOL (Schrödinger, LLC). The Fc regions in the 10 best fit IgG4 models were superimposed using the align function within PyMOL. To dock the crystal structures for the Fc region and the C1q head (19), the web server algorithm PatchDock (version beta 1.3) was used to include specified residues as potential binding sites, and then the output was refined using FireDock from the same web site (44).

##### Protein Data Bank Accession Numbers

The three sets of 10 best fit models are currently available as supplemental material. They have been deposited in the Protein Data Bank under accession codes 4PTO (IgG4(Ser^222^) in PBS-137), 4PTQ (IgG4(Ser^222^) in PBS-137 in ^2^H_2_O), and 4PTR (IgG4(Pro^222^) in PBS-137).

## RESULTS

### 

#### 

##### Purification of Human IgG4

Wild-type IgG4(Ser^222^) and its mutant IgG4(Pro^222^) were subjected to gel filtration to ensure monodispersity immediately prior to the ultracentrifugation and scattering experiments. Both molecules eluted as a symmetric main peak at 16 ml (supplemental Fig. S3) and showed a single band between 200 and 116 kDa in non-reducing SDS-PAGE that corresponds to the expected masses of 147.0 and 149.5 kDa for intact IgG4(Ser^222^) and IgG4(Pro^222^), respectively. Under reducing conditions, the heavy chains for both IgG4 molecules were observed at an apparent molecular mass of 55 kDa, and their light chains were between 31 and 21.5 kDa, both as expected (supplemental Fig. S3).

##### Analytical Ultracentrifugation of Human IgG4

**FIGURE 1.**
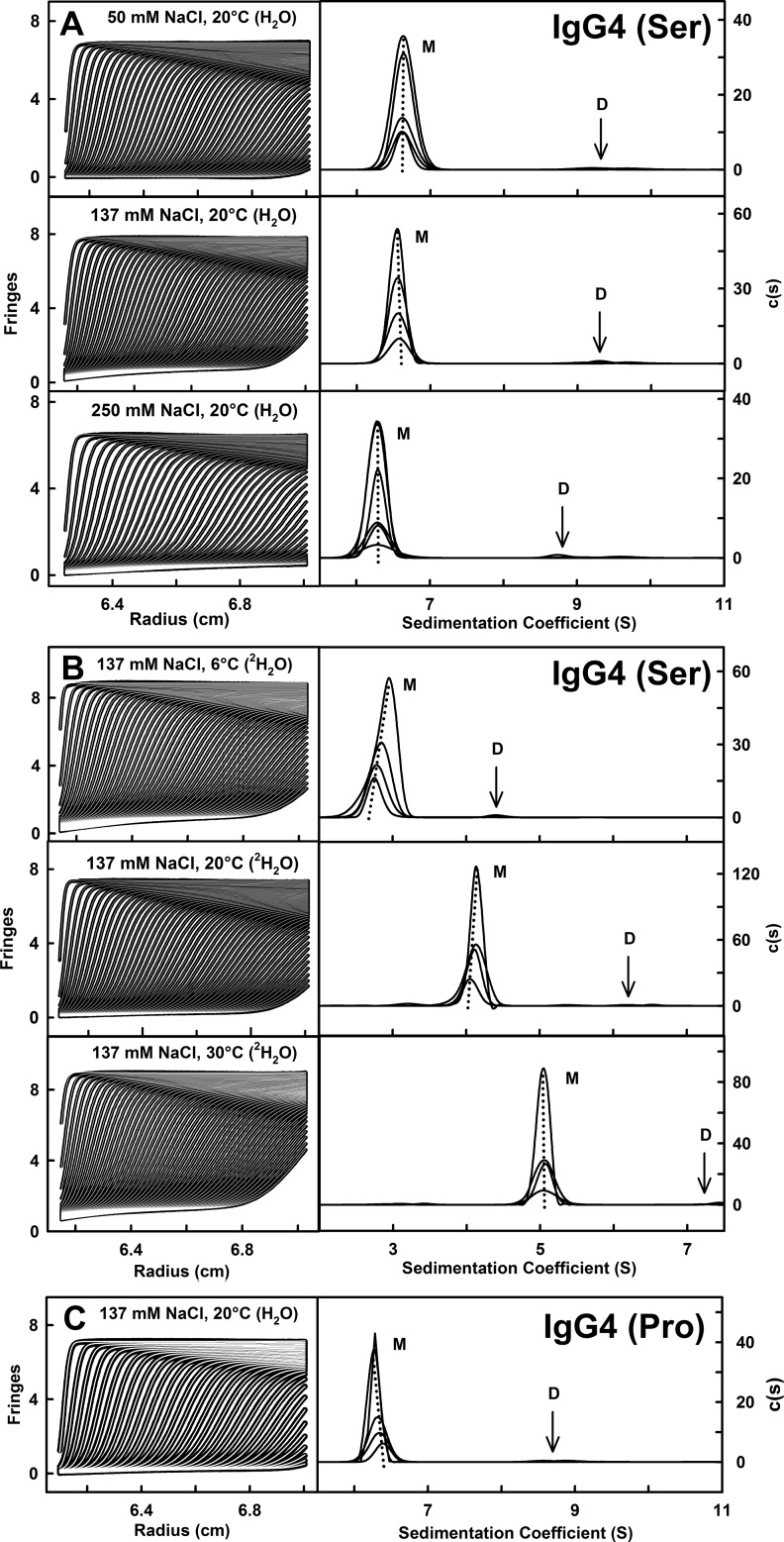
**Sedimentation velocity analyses of human IgG4.** *A*, on the *left*, the experimentally observed sedimentation boundaries for IgG4(Ser^222^) in PBS-50, PBS-137, and PBS-250 in H_2_O were recorded at a rotor speed of 40,000 rpm. *B*, IgG4(Ser^222^) was measured at 6, 20, and 30 °C in PBS-137 in ^2^H_2_O at a rotor speed of 40,000 rpm. *C*, IgG4(Pro^222^) in PBS-137 in H_2_O was also measured at 40,000 rpm. Fifty boundaries (*black outlines*) are shown from as many as 700 scans at intervals of every *e.g.* 14th scan for clarity. The SEDFIT fits are shown as *white lines*. On the *right*, the observed *s* values in the corresponding size distribution analyses *c*(*s*) revealed a monomer (*M*) peak at *s*_20,*w*_^0^ values of ∼6.8 S for IgG4(Ser^222^) and 6.6 S for IgG4(Pro^222^) in the H_2_O buffers together with a minor dimer peak (*D*) at about 9 S. The observed *s* values in ^2^H_2_O buffers are shifted to lower S values.

Sedimentation velocity experiments examined the size and shape of IgG4(Ser^222^) between 0.5 and 8 mg/ml and IgG4(Pro^222^) between 0.5 and 2.5 mg/ml. The SEDFIT analyses in different NaCl buffers in H_2_O and at different temperatures in ^2^H_2_O buffers involved fits of as many as 700 scans, and the good agreement between the experimental boundary scans and fitted lines is clear (Fig. 1, *A–C*). A major monomer peak was observed at *s*_20,*w*_^0^ values of 6.8 S for IgG4(Ser^222^) and 6.6 S for IgG4(Pro^222^). These *s*_20,*w*_^0^ values are consistent with the range of values (6.2–6.9 S) reported previously for human IgG4 (20, 45–48). Accordingly both IgG4(Ser^222^) and IgG4(Pro^222^) were predominantly monomeric in solution and were accompanied by a minor dimer peak.

From the *c*(*s*) analyses, the molecular masses of the monomer peak for IgG4(Ser^222^) were reported as 144 (PBS-50), 145 (PBS-137), and 141 kDa (PBS-250) in light water and 171 (PBS-137 at 6 °C), 149 (PBS-137 at 20 °C), and 145 kDa (PBS-137 at 30 °C) in heavy water. These agree well with the composition-calculated mass of 147 kDa with the exception of PBS-137 in heavy water at 6 °C. However, for PBS-137 in heavy water at 6 °C, a reaction boundary was observed from the concentration dependence of its monomer peak (Fig. 1*B*). The molecular mass of the IgG4(Pro^222^) monomer peak was measured as 143 kDa (PBS-137) in agreement with the composition-calculated mass of 150 kDa.

**FIGURE 2.**
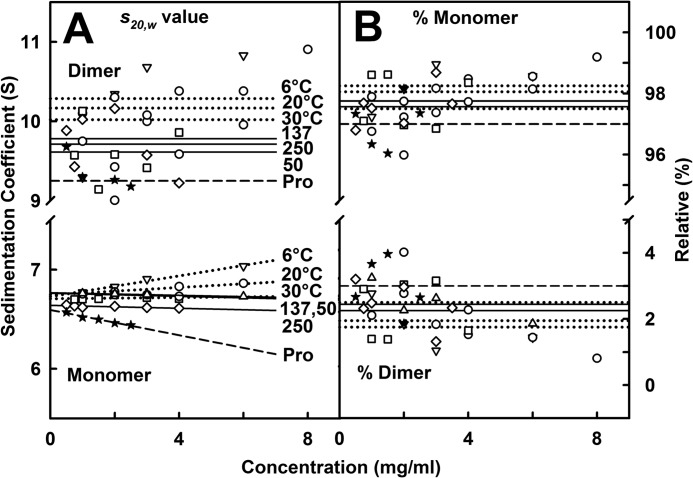
**Concentration dependence of the IgG4 sedimentation analyses.** *A*, the concentration dependence of the *s*_20,_*_w_* values for the IgG4 monomer and dimer peaks of Fig. 1 are shown as a function of IgG4(Ser^222^) concentration. The mean *s*_20,_*_w_* values are shown as *solid lines* for PBS-50 (□), PBS-137 (○), and PBS-250 (♢) in H_2_O buffer at 20 °C and as *dotted lines* for 6 (▵), 20 (○), and 30 °C (▿) for PBS-137 at 20 °C in ^2^H_2_O buffer. Data for IgG4(Pro^222^) (★) in PBS-137 in H_2_O at 20 °C are shown as two *dashed lines. B*, the mean percentages of the major monomer and minor dimer forms from integration of the *c*(*s*) analyses are shown for PBS-50, PBS-137, and PBS-250 at 20 °C (*solid lines*) and at 6, 20, and 30 °C for PBS-137 in ^2^H_2_O (*dotted lines*). Data for IgG4(Pro^222^) (★) in PBS-137 in H_2_O at 20 °C are shown as two *dashed lines*.

The apparent sedimentation rates of the IgG4 monomer depended on the buffer composition and temperature (Fig. 1, *A–C*). The monomer *s*_20,_*_w_* value decreased slightly with rotor speed. For IgG4(Ser^222^), the monomer *s*_20,*w*_^0^ value was 6.76 S for 40,000 rpm, decreasing to 6.68 S for 50,000 rpm for PBS-137 at 20 °C. For IgG4(Pro^222^), the monomer *s*_20,*w*_^0^ value was 6.64 S for 30,000 rpm, decreasing to 6.59 S for 40,000 rpm, 6.56 S for 50,000 rpm, and 6.53 S for 60,000 rpm. All the IgG4 data reported in this study are for 40,000 rpm. Extrapolation of the *s*_20,_*_w_* values to zero concentration gave monomer *s*_20,*w*_^0^ values of 6.71, 6.76, and 6.64 S for the PBS-50, PBS-137, and PBS-250 buffers, respectively, in light water (Fig. 2*A*). In PBS-137 in heavy water, the monomer sedimented at apparent rates of 2.75, 4.07, and 5.07 S at 6, 20, and 30 °C, respectively (Fig. 1*B*). When corrected for the buffer density and viscosity of heavy water, *s*_20,*w*_^0^ values of 6.49, 7.22, and 7.35 S, respectively, were obtained. Given that the partial specific volume *v* for proteins is affected by the hydration shell (27, 40) and that the hydration shell for heavy water has a higher mass than that for light water, the *v* values will be reduced in 100% ^2^H_2_O. Additionally, the hydration shell is affected by temperature as shown by the large difference between the *s*_20,*w*_^0^ values at 6 and 30 °C. Thus *v* values of 0.734, 0.716, and 0.713 ml/g were used for 6, 20, and 30 °C, respectively, in place of 0.728 ml/g, and these gave final monomer *s*_20,*w*_^0^ values of 6.76 S for each temperature, which are the same as that for PBS-137 in light water. For IgG4(Pro^222^), the *s*_20,*w*_^0^ value was 6.59 S for PBS-137 at 20 °C, which is not significantly different from that for IgG4(Ser^222^).

The *c*(*s*) analyses for IgG4(Ser^222^) revealed a minor dimer peak at *s*_20,*w*_^0^ values between 9 and 10 S in the size distribution analyses *c*(*s*) (Figs. 1 and 2). The molecular masses in light water were 259 ± 16 (PBS-50), 270 ± 11 (PBS-137), and 264 ± 11 kDa (PBS-250) and slightly larger in heavy water at 315 ± 24 (PBS-137 at 6 °C), 286 ± 36 (PBS-137 at 20 °C), and 281 ± 9 kDa (PBS-137 at 30 °C). These masses are comparable with the expected value of 294 kDa for the IgG4(Ser^222^) dimer. IgG4(Pro^222^) also showed a small dimer peak of size 2–3% with a *s*_20,*w*_^0^ value of 9.25 ± 3.0 S and a molecular mass of 276 ± 15 kDa. This agreed well with the predicted mass of 300 kDa for its dimer. The monomer and dimer *c*(*s*) peaks were integrated. The amount of dimer did not alter with concentration or buffer composition (Fig. 2*B*) and totaled between 1 and 3% dimer. The monomer/dimer ratio corresponded to weak dissociation constants (*K_D_*) in a range of 600–3000 μm. In comparison, dimer formation for rabbit IgG was noticeably higher with the corresponding *K_D_* values ranging from 70 μm for PBS-137 at 20 °C in heavy water to 350 μm in PBS-137 at 30 °C (19). The *s*_20,*w*_^0^ values were similar in light water at 9.61 ± 0.34 (PBS-50), 9.78 ± 0.32 (PBS-137), and 9.71 ± 0.36 S (PBS-250) at 20 °C. They were slightly larger but similar at 10.27 ± 0.70, 10.17 ± 0.81, and 10.02 ± 0.23 S at 6, 20, and 30 °C, respectively, in PBS-137 in heavy water.

##### X-ray and Neutron Scattering of Human IgG4

The solution structure of IgG4 was jointly analyzed by both x-ray and neutron scattering for reproducibility. X-rays in light water buffers monitor the hydration shell as well as the protein structure, whereas neutrons in heavy water buffers monitor the unhydrated structure (see below).

**FIGURE 3.**
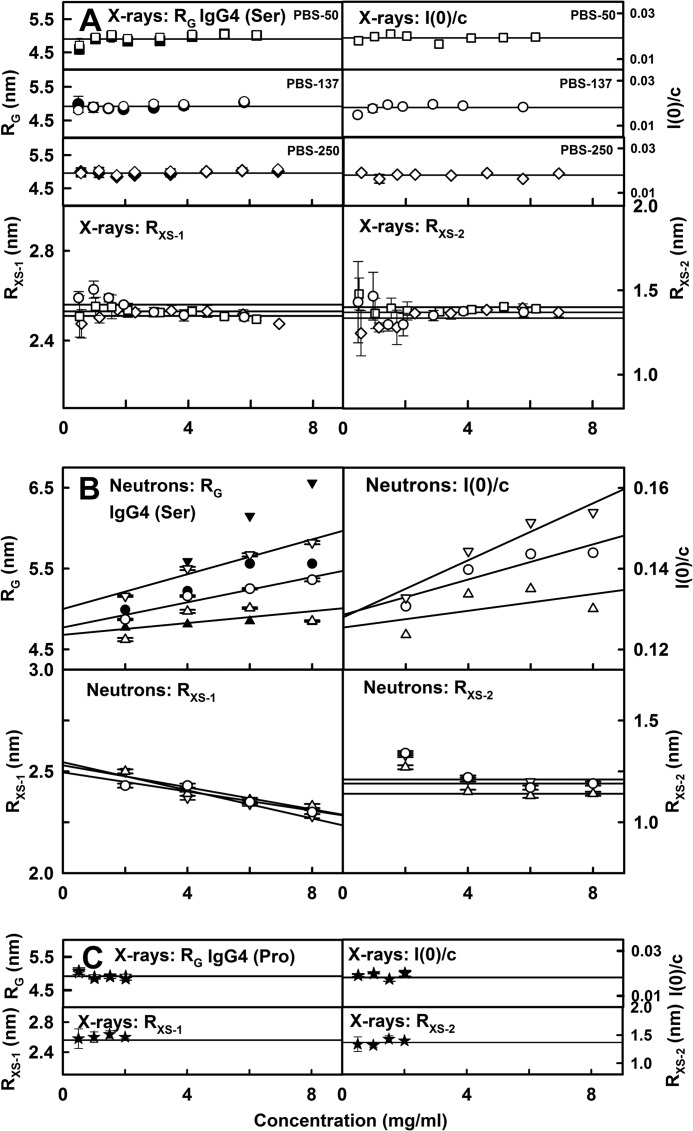
**Concentration and temperature dependences of the x-ray and neutron Guinier analyses.** *A*, the x-ray Guinier values for IgG4(Ser^222^) were measured in quadruplicate and averaged and are shown as the mean ± S.D. All the *lines* show the mean value. *Error bars* representing S.D. are shown only when larger than the symbol. The x-ray *R_g_* values for IgG4(Ser^222^) are shown for PBS-50 (□ and ■), PBS-137 (○ and ●), and PBS-250 (♢ and ♦). The *open symbols* correspond to the Guinier values, and the *filled symbols* correspond to the *P*(*r*) values. The corresponding x-ray *I*(0)/*c*, *R*_XS-1_, and *R*_XS-2_ values are likewise shown for the three buffers. *B*, the neutron values correspond to single measurements in PBS-137 in ^2^H_2_O. The *lines* correspond to linear regression fits. The *R_g_* values at 6 (▿ and ▾), 20 (○ and ●), and 37 °C (▵ and ▴) are shown with the *open symbols* corresponding to the Guinier values and *filled symbols* corresponding to the *P*(*r*) values. The corresponding *I*(0)/*c*, *R*_XS-1_, and *R*_XS-2_ values are likewise shown. The fitted *line* shown for *R*_XS-2_ is the mean value. *C*, the corresponding x-ray Guinier values for IgG4(Pro^222^) (★) are shown in the same view as that of *A*.

X-rays were most effective for looking at IgG4 at 20 °C in three different NaCl concentrations. X-ray data collection of IgG4(Ser^222^) was carried out between 0.48 and 6.91 mg/ml using time frame analyses to ensure the absence of radiation damage effects. Guinier analyses resulted in high quality linear plots in three distinct regions of the *I*(*Q*) curves, as expected for antibodies, from which the *R_g_*, *R*_XS-1_, and *R*_XS-2_ values were obtained within satisfactory *Q*·*R_g_* and *Q*·*R*_XS_ limits (supplemental Fig. S4A and Table 1). The x-ray *R_g_* values for IgG4(Ser^222^) in PBS-50, PBS-137, and PBS-250 showed no concentration dependence with mean values of 4.90, 4.92, and 4.96 nm, respectively (Fig. 3*A*). The *I*(0)/*c* values for IgG4, which were similar at 0.0192, 0.0181, and 0.0179, respectively, also showed no concentration dependence (Fig. 3*A*). Each of the *R*_XS-1_ and *R*_XS-2_ values were unchanged among PBS-50, PBS-137, and PBS-250 with mean *R*_XS-1_ values of 2.53, 2.56, and 2.51 nm, respectively, and mean *R*_XS-2_ values of 1.40, 1.37, and 1.33 nm, respectively. These values agree with the *R_g_*, *R*_XS-1_, and *R*_XS-2_ values of 4.99, 2.56, and 1.40 nm, respectively, for this same human IgG4(Ser^222^) molecule in PBS-137 (20). For comparison with IgG4(Ser^222^), IgG4(Pro^222^) was studied between 0.5 and 2.0 mg/ml in PBS-137 (supplemental Fig. S4C). The x-ray *R_g_* values showed no concentration dependence with a mean value of 4.90 nm (Fig. 3*C*). Similarly, the *I*(0)/*c* values for IgG4(Pro^222^) were 0.0191, showing no concentration dependence and in agreement with those for IgG4(Ser^222^) (Fig. 3*C*). The mean *R*_XS-1_ and *R*_XS-2_ values were unchanged at 2.60 and 1.37 nm, respectively (Fig. 3*C*).

**TABLE 1:** **Modeling searches of the x-ray and neutron scattering and sedimentation coefficient data for human IgG4**

	Filter	Models	Spheres*^a^*	*R_g_**^b^*	*R*_XS-1_	*R*_XS-2_	*D*_max_	*R*-factor	*s*_20,*w*_^0^*^c^*
				*nm*	*nm*	*nm*	*nm*	%	*S*
**X-ray data IgG4(Ser^222^) in PBS-137**									
IgG4 x-ray models	None	20,000	1047–1554	3.05–7.01	0.07–3.32	0.05–2.85	n.a.	n.a.	n.a.
X-ray fit, 0.48 mg/ml	*R_g_*, *R*_XS_, spheres	10	1455–1478	4.74–4.80	2.48–2.59	1.53–1.64	n.a.	4.0–4.1	6.88–6.99; 6.57–6.83
Experimental data	n.a.*^d^*	n.a.	n.a.	4.99 ± 0.23; 4.81 ± 0.06	2.59 ± 0.03	1.43 ± 0.24	16	n.a.	6.76
X-ray fit, 0.96 mg/ml	*R_g_*, *R*_XS_, spheres	10	1460–1512	4.72–4.90	2.56–2.61	1.52–1.61	n.a.	3.1–3.2	6.83–6.93; 6.55–6.92
Experimental data	n.a.	n.a.	n.a.	4.89 ± 0.15; 4.90 ± 0.09	2.63 ± 0.04	1.47 ± 0.14	16	n.a.	6.76
X-ray fit, 1.93 mg/ml	*R_g_*, *R*_XS_, spheres	10	1461–1501	4.71–4.93	2.50–2.66	1.24–1.34	n.a.	2.5–2.6	6.81–6.95; 6.58–6.79
Experimental data	n.a.	n.a.	n.a.	4.83 ± 0.02; 4.93 ± 0.01	2.56 ± 0.01	1.30 ± 0.07	16	n.a.	6.76
X-ray fit, 3.86 mg/ml	*R_g_*, *R*_XS_, spheres	10	1451–1483	4.87–4.98	2.50–2.61	1.34–1.43	n.a.	2.7–2.9	6.76–6.91; 6.43–6.94
Experimental data	n.a.	n.a.	n.a.	4.94 ± 0.11; 4.98 ± 0.06	2.51 ± 0.03	1.38 ± 0.02	16	n.a.	6.76
X-ray fit, 5.79 mg/ml	*R_g_*, *R*_XS_, spheres	10	1451–1496	4.82–4.98	2.48–2.61	1.34–1.45	n.a.	2.8–3.0	6.76–6.93; 6.42–6.94
Experimental data	n.a.	n.a.	n.a.	5.04 ± 0.05; 5.07 ± 0.04	2.51 ± 0.02	1.37 ± 0.03	16	n.a.	6.76

**Neutron data IgG4(Ser^222^) in PBS-137**									
IgG4 neutron models	None	20,000	862–1177	2.91–5.04	0.18–2.73	0.33–2.47	n.a.	n.a.	n.a.
Neutron fit, 0 mg/ml	*R_g_*, *R*_XS_, spheres	10	1131–1167	4.74–4.86	2.50–2.55	1.14–1.24	n.a.	2.5–2.7	7.15–7.37; 6.38–6.71
Experimental data	n.a.	n.a.	n.a.	4.77	2.49	1.19	16	n.a.	6.76

**X-ray data IgG4(Pro^222^) in PBS-137**									
X-ray fit, 0.5 mg/ml	*R_g_*, *R*_XS_, spheres	10	1466–1494	4.92–5.04	2.61–2.69	1.38–1.48	n.a.	3.6–3.7	6.72–6.82; 6.23–6.69
Experimental data	n.a.	n.a.	n.a.	4.97 ± 0.02; 5.02	2.58 ± 0.13	1.34 ± 0.13	15	n.a.	6.59
X-ray fit, 1.0 mg/ml	*R_g_*, *R*_XS_, spheres	10	1402–1473	4.75–4.93	2.52–2.63	1.29–1.40	n.a.	3.4–3.6	6.84–7.02; 6.60–6.84
Experimental data	n.a.	n.a.	n.a.	4.85 ± 0.01; 4.90	2.60 ± 0.07	1.32 ± 0.03	15	n.a.	6.59
X-ray fit, 1.5 mg/ml	*R_g_*, *R*_XS_, spheres	10	1429–1476	4.75–4.97	2.59–2.64	1.33–1.44	n.a.	3.0–3.2	6.85–7.01; 6.48–6.84
Experimental data	n.a.	n.a.	n.a.	4.84 ± 0.01; 4.93	2.64 ± 0.05	1.43 ± 0.06	15	n.a.	6.59
X-ray fit, 2.0 mg/ml	*R_g_*, *R*_XS_, spheres	10	1429–1482	4.75–4.97	2.56–2.64	1.36–1.45	n.a.	3.0–3.2	6.78–7.01; 6.46–6.86
Experimental data	n.a.	n.a.	n.a.	4.82 ± 0.01; 4.92	2.60 ± 0.02	1.40 ± 0.03	15	n.a.	6.59

*^a^* The optimum number *n* of hydrated (x-rays) and unhydrated (neutrons) spheres predicted from the sequence was 1468 and 1142, respectively.

**FIGURE 4.**
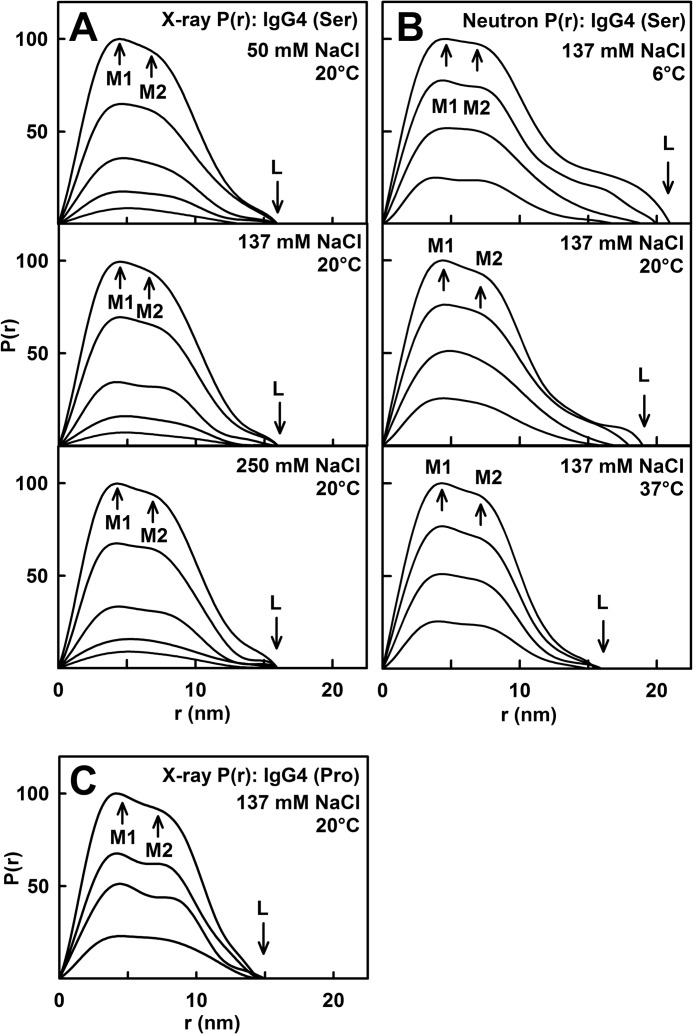
**X-ray and neutron distance distribution analyses *P*(*r*) for IgG4.** The peak maxima at M1 and M2 and the maximum length at *L* are indicated by *arrows. A*, the x-ray *P*(*r*) curves for IgG4(Ser^222^) in PBS-50, PBS-137, and PBS-250 are shown at concentrations between 0.5 and 6 mg/ml. *B*, the neutron *P*(*r*) curves for IgG4(Ser^222^) in PBS-137 in ^2^H_2_O buffer at 6, 20, and 37 °C are shown for concentrations between 2 and 8 mg/ml. *C*, the x-ray *P*(*r*) curves for IgG4(Pro^222^) in PBS-137 are shown for concentrations between 0.5 and 2 mg/ml.

**FIGURE 5.**
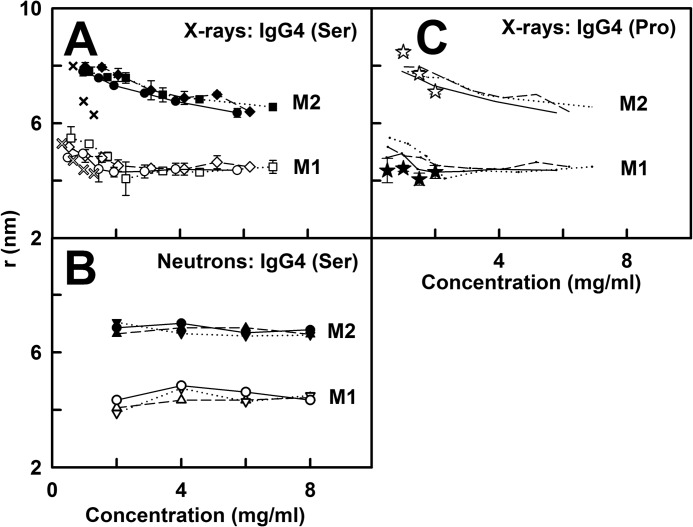
**Concentration dependence of the *P*(*r*) analyses for IgG4.** *A*, the x-ray values of the *P*(*r*) maxima M1 and M2 are shown for IgG4(Ser^222^) in PBS-50 (M1, ♢; M2, ♦; *dashed line*), PBS-137 (M1, ○; M2, ●; *solid line*), and PBS-250 (M1, □; M2, ■; *dotted line*). These are compared with the 2010 values (20) for IgG4(Ser^222^) in PBS-137 (M1, *open* ×; M2, *closed* ×). *Error bars* represent S.D. *B*, the neutron values for IgG4(Ser^222^) are shown for 6 (M1, ▿; M2, ▾; dotted line), 20 (M1, ○; M2, ●; *solid line*), and 37 °C (M1, ▵; M2, ▴; *dashed line*). *C*, the pairs of x-ray M1 and M2 values for IgG4(Pro^222^) in PBS-137 (M1, ★; M2, ☆) are compared with the data for IgG4(Ser^222^) depicted as *lines* from *A*.

*^b^* The first experimental value is from the Guinier *R_g_* analysis (Fig. 4), and the second is from the GNOM *P*(*r*) analysis (Fig. 5).

*^c^* The first modeled value corresponds to that from HYDRO, and the second is that from HYDROPRO.

*^d^* n.a., not applicable.

Neutron scattering studied the unhydrated protein structure in which the hydration shell is almost invisible in heavy water (40). Neutrons were most useful for temperature studies in PBS-137 because temperature-dependent conditions were less accessible by x-ray scattering. IgG4(Ser^222^) in 100% ^2^H_2_O buffer was analyzed between 2.0 and 8.0 mg/ml. The Guinier analyses revealed high quality linear fits for the same three parameters, *R_g_*, *R*_XS-1_, and *R*_XS-2_, as described above for x-rays (supplemental Fig. S4B). The neutron *R_g_* values increased with increasing concentration from 5.16 to 5.82 nm at 6 °C, from 4.87 to 5.36 nm at 20 °C, and from 4.62 to 4.85 nm at 37 °C (Fig. 3*B*). These *R_g_* changes were comparatively large, indicating that heavy water promoted IgG4 dimer formation. Because the neutron Guinier fits go to smaller *Q* values than the x-ray Guinier fits, the neutron fits were more sensitive to dimer formation than the x-ray fits. Extrapolations to zero concentration resulted in *R_g_* values of 4.99 (6 °C), 4.77 (20 °C), and 4.67 nm (37 °C). The corresponding *I*(0)/*c* values also increased (Fig. 3*B*). The neutron *R*_XS-1_ values decreased slightly with concentration from 2.50 to 2.28 nm at 6 °C, from 2.43 to 2.30 nm at 20 °C, and from 2.50 to 2.33 nm at 37 °C (Fig. 3*B*). The *R*_XS-2_ values showed no concentration dependence with mean values of 1.23, 1.23, and 1.17 nm at 6, 20, and 37 °C, respectively (Fig. 3*B*). The neutron *R_g_*, *R*_XS-1_, and *R*_XS-2_ values were slightly smaller than the x-ray values, such as that of 4.92 nm for the *R_g_* value in PBS-137, and this reduction is attributed to the near invisibility of the surface hydration shell in heavy water (40) as well as the high negative solute-solvent contrast difference.

The distance distribution function *P*(*r*) provides structural information on IgG4 in real space. The x-ray *P*(*r*) analyses gave *R_g_* and *I*(0)/*c* values for IgG4(Ser^222^) that were similar to those from the x-ray Guinier analyses, showing that the two analyses were self-consistent (Fig. 3*A*, *filled* and *open symbols*). The maximum length *L* of IgG4 was determined from the value of *r* when the *P*(*r*) curve intersects zero to be 16 nm for PBS-50, PBS-137, and PBS-250 (Fig. 4*A*). The maxima in the *P*(*r*) curves correspond to the most frequently occurring interatomic distances within the structure. For IgG4(Ser^222^), two peaks, M1 and M2, were identified in many *P*(*r*) curves except for that of the 0.5 mg/ml *P*(*r*) curve where only M1 was identified. The M1 peak corresponds mostly to distances within each Fab and Fc region, whereas the M2 peak corresponds mostly to distances between pairs of Fab and Fc regions. No buffer dependence in the positions of peaks M1 and M2 was observed. However, the positions of the M2 peaks depended on the IgG4 concentration below 2 mg/ml (Fig. 5*A*). The more extensive data sets of this study confirmed this dependence seen previously with more limited data (20). The concentration dependence was attributed to a rearrangement of the Fab regions in IgG4(Ser^222^) in which a 1–2-nm change in the average separation between the Fab-Fc pairs was deduced (20). At concentrations above 2 mg/ml, our new data show that this effect is absent with M1 and M2 being at ∼4 and 7 nm, respectively (Fig. 5*A*). The IgG4(Pro^222^) mutant also showed two M1 and M2 peaks with a similar pronounced shift in the M2 peak (but an unchanged M1 peak) with change in concentration, and the *L* value was unchanged at 15 nm (Figs. 4*C* and 5*C*).

The neutron *P*(*r*) analyses reflected IgG4 dimer formation in heavy water. The *R_g_* and *I*(0)/*c* values for IgG4(Ser^222^) at 6, 20, and 37 °C increased with increasing concentration (Fig. 3*B*). This was attributed to dimer formation. This result is consistent with the sedimentation velocity experiments where a reaction boundary between monomer and dimer was observed in heavy water at 6 and 20 °C (Fig. 1*B*) but not in light water. The neutron *L* values were between 17 and 21 nm at 6 °C, 16 and 19 nm at 20 °C, and 15 and 16 nm at 37 °C (Fig. 4*B*). Thus the greater proportion of dimer at lower temperatures resulted in increased dimensions. Because low amounts of dimer are present, the maximum length of the dimer is probably greater than 21 nm. The two peaks M1 and M2 were again identified at ∼4 and 7 nm, respectively, in the neutron *P*(*r*) curves (Fig. 5*B*). The positions of M1 and M2 were unchanged between 2 and 8 mg/ml in agreement with the x-ray *P*(*r*) data.

##### Starting Model for the Human IgG4 Scattering Fits

The human IgG4 homology models were created from crystal structures for B72.3 Fab (36) and human IgG1 Fc (37). Details of the sequence alignments, target and template sequence identities, and structure validations are described elsewhere (20). The previous modeling of hinge libraries with 10,000 conformations based on asymmetric IgG4 structures was now extended to include libraries based on symmetric IgG4 structures (“Experimental Procedures”). The full hinge is formally defined by the residues ^213^ESKYGPPCPSCPAPEFLGGP^232^ (22) in which the Fab region ends at Val^212^ and the Fc region starts at Ser^233^ (supplemental Figs. S1 and S2). The IgG4 hinge contains six Pro residues and two Cys^220^ and Cys^223^ disulfide bridges. The asymmetric modeling considered only the upper hinge, ^212^VESKYGPPC^220^, with Val^212^ and Cys^220^ acting as tethers. As this hinge is located asymmetrically relative to the Fc region, these 10,000 models do not have 2-fold symmetry. However, the two interchain disulfide bonds are intact. The symmetric modeling considered the upper, middle, and lower hinge, and this resulted in a 21-residue peptide, ^212^VESKYGPPCPSCPAPEFLGGP^232^. Because the two Pro^232^ residues were located in the middle of the Fc region, this approach generated both symmetric and asymmetric models. For these 10,000 further models, the Cys^220^ and Cys^223^ interchain disulfide bonds were not kept intact.

##### Conformational Searches for the Human IgG4 Solution Structure

**FIGURE 6.**
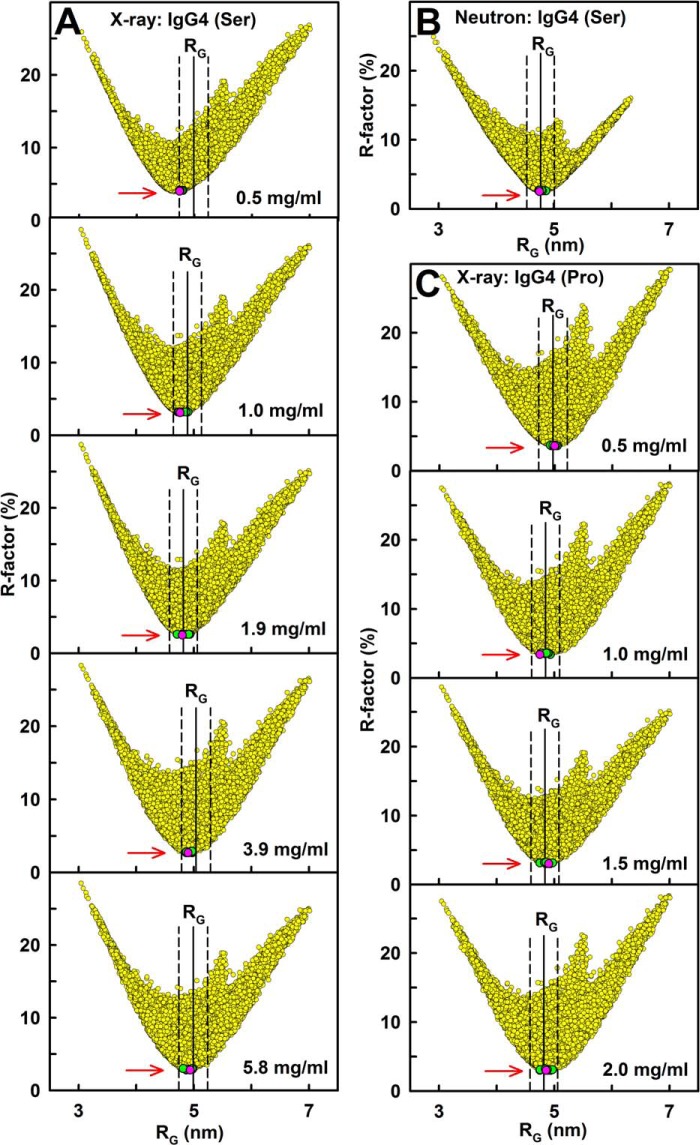
**Constrained modeling analysis for IgG4.** The 20,000 goodness-of-fit *R*-factors are compared with the x-ray and neutron *R_g_* values for the conformationally randomized IgG4(Ser^222^) models. The 20,000 asymmetric and symmetric models are shown in *yellow*. The 10 best fit models with the lowest *R*-factors are shown in *green* of which the best fit model is shown in *pink*. The experimentally observed *R_g_* values are shown by *vertical solid lines* to follow the Guinier *R_g_* values (Table 1) with ±5% error ranges shown as *dashed lines. A*, the hydrated x-ray models are compared with the x-ray curves for IgG4(Ser^222^) in PBS-137 for each of five concentrations between 0.5 and 6 mg/ml. *B*, the unhydrated neutron models are compared with the neutron curve for IgG(Ser^222^) in PBS-137 in ^2^H_2_O after linear extrapolation to 0 mg/ml. *C*, the hydrated x-ray models are compared with the x-ray curves for IgG4(Pro^222^) in PBS-137 for each of four concentrations between 0.5 and 2 mg/ml.

To model the IgG4(Ser^222^) and IgG4(Pro^222^) solution structures, 20,000 conformationally randomized IgG4 structures were created from the Fab and Fc structures starting from four conformational libraries of randomized hinge peptides of lengths 2.2–3.1 and 3.1–3.15 nm (asymmetric) and 5.0–7.0 and 7.0–7.35 nm (symmetric) (“Experimental Procedures”). Each modeled scattering curve was compared with the experimental x-ray and neutron scattering curves. The nine x-ray curves included IgG4(Ser^222^) in PBS-137 at concentrations of 0.48, 0.96, 1.93, 3.86, and 5.79 mg/ml and IgG4(Pro^222^) in PBS-137 at concentrations of 0.5, 1.0, 1.5, and 2.0 mg/ml. These curve fits investigated whether molecular models could rationalize the conformational change seen in IgG4 at below 2 mg/ml for the wild-type and mutant forms of IgG4 (Fig. 5, *A* and *C*). Because IgG4 dimerizes in heavy water, neutron curve fits were based on linear extrapolation of the four experimental curves for IgG4(Ser^222^) to zero concentration (Fig. 3*B*). This curve fit examined a single conformation for the IgG4 monomer only. The 10 fit searches were assessed in *R*-factor *versus R_g_* graphs (Fig. 6). In all 10 searches, the occurrence of a single minimum in the *R*-factor values identified a single family of solution structures for IgG4 starting from a wide range of orientations and translations of the two Fab and Fc regions. The lowest *R*-factors in the 20,000 curve fits corresponded to modeled *R_g_* values close to the experimental *R_g_* values as desired.

Filters were used to identify the 10 best fit models for each search. (i) A ±5% filter for steric overlap eliminated models in which the Fab and Fc regions sterically overlapped with each other because of inappropriate hinge conformations. To match the composition-calculated volume of IgG4, sphere models needed a minimum number *n* of 1468 spheres for the hydrated x-ray models and 1142 spheres for the unhydrated neutron models. (ii) A ±5% filter for the modeled *R_g_* values (calculated from the same *Q* ranges used for the experimental analyses) identified the models that agreed best with the experimental x-ray or neutron *R_g_* values. (iii) The models that passed the *n* and *R_g_* filters were arranged in order of their lowest *R*-factors. The resulting 10 best fit models for IgG4 were found as a single cluster at the *R*-factor minimum in each of the 10 searches (Fig. 6, *green*).

The two interchain disulfide bonds at Cys^220^ and Cys^223^ were explicitly conserved in the asymmetric models. For the symmetric models, the pairs of Cys^220^ and Cys^223^ residues may no longer be proximate. Their α-carbon separations were 1.02–3.29 nm for Cys^220^ and 1.20–2.43 nm for Cys^223^ in IgG4(Ser^222^) by x-rays, 0.75–2.18 nm for Cys^220^ and 0.98–1.99 nm for Cys^223^ in IgG4(Pro^222^) by x-rays, and 2.02 nm for Cys^220^ and 1.98 nm for Cys^223^ for IgG4(Ser^222^) by neutrons. These α-carbon separations were comparable with an expected α-carbon separation of 0.4–0.75 nm between two bridged Cys residues (49).

**FIGURE 7.**
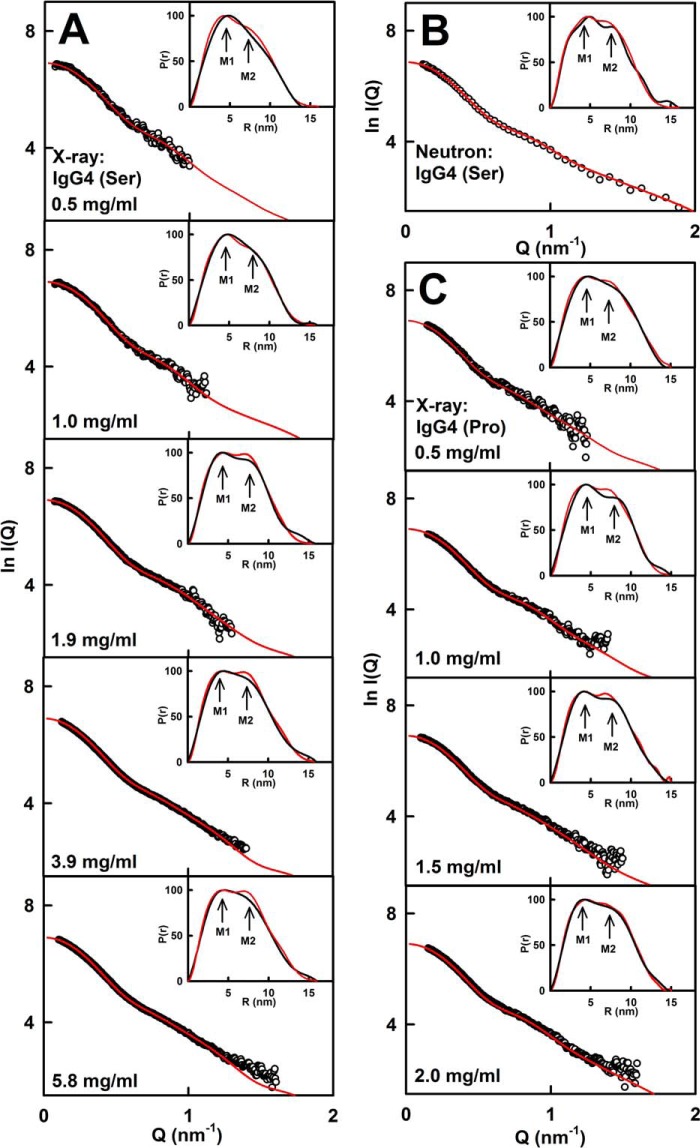
**X-ray and neutron scattering curve fits for the best fit IgG4 models.** *A*, IgG4(Ser^222^) fits in PBS-137 by x-ray scattering. *B*, IgG4(Ser^222^) fits in PBS-137 in ^2^H_2_O extrapolated to zero concentration by neutron scattering. *C*, IgG4(Pro^222^) fits in PBS-137 by x-ray scattering. The experimental data at 20 °C are indicated by *circles* (*black*), and the modeled best fit scattering curve is indicated by the *continuous line* (*red*). The *insets* correspond to the experimental (*black*) and best fit modeled (*red*) curves in which the M1 and M2 values are indicated by *arrows*.

**FIGURE 8.**
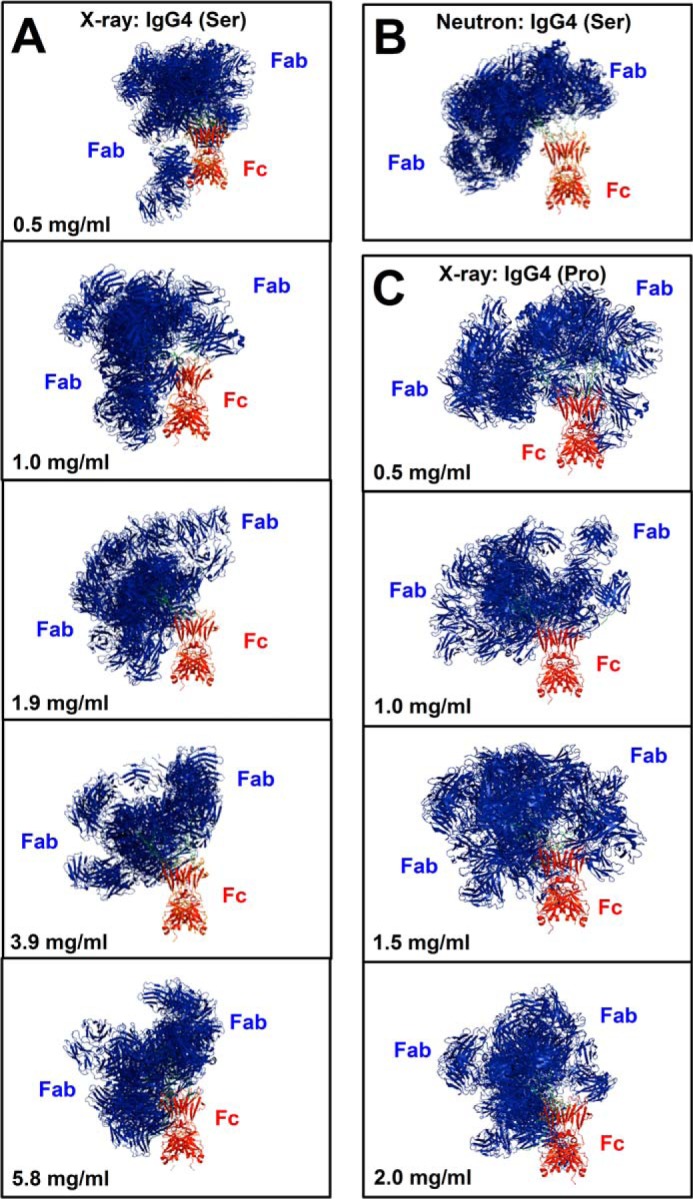
**The best fit IgG4 models.** The 10 best fit models from each analysis are shown superimposed upon their Fc region (*red*). *A*, IgG4(Ser^222^) in PBS-137 (x-rays). *B*, IgG4(Ser^222^) in PBS-137 in ^2^H_2_O (neutrons). *C*, IgG4(Pro^222^) in PBS-137 (x-rays).

The best fit modeled curves showed good visual scattering *I*(*Q*) and *P*(*r*) curve fits in all 10 cases with the experimental curves (Fig. 7). For each case, the *R_g_* values for the 10 best fit models were within error of the experimental values (Table 1). The 10 sets of models in Fig. 8 generally displayed asymmetric arrangements of the two Fab regions compared with the Fc region. Although IgG4(Ser^222^) showed slightly higher proportions of asymmetric structures compared with the asymmetric and symmetric arrangements seen for IgG4(Pro^222^), there were no significant differences between the wild-type and mutant structures. Unlike our previous study (20), no gradual continuum between the models at different concentrations was now seen. Surveys of the distances between the centers of the Fab and Fc regions in the best fit IgG4(Ser^222^) x-ray and neutron models and in the IgG4(Pro^222^) x-ray models (Figs. 6 and 8) showed similar distributions (supplemental Fig. S5). The x-ray *R*-factor values for the best fit IgG4 models (Fig. 6, *pink*) were acceptable at 2.5–4.0% for IgG4(Ser^222^) and 3.0–3.6% for IgG4(Pro^222^) (Table 1). The neutron *R*-factor values were acceptable at 2.5–2.7%. These *R*-factor values are comparable with those from other similar modeling fits (43).

##### Sedimentation Coefficient Modeling of Human IgG4

The *s*_20,*w*_^0^ values of each of the nine sets of scattering best fit hydrated IgG4 models were calculated for comparison with the experimental values of 6.76 S for IgG4(Ser^222^) and 6.59 S for IgG4(Pro^222^) (Fig. 2*A*). For the best fit hydrated sphere models, the *s*_20,*w*_^0^ values were 6.76–6.99 and 6.72–7.02 S for IgG4(Ser^222^) and IgG4(Pro^222^), respectively, using HYDRO (Table 1). For the best fit molecular models, the *s*_20,*w*_^0^ values were 6.42–6.94 and 6.23–6.84 S for IgG4(Ser^222^) and IgG4(Pro^222^), respectively, using HYDROPRO (Table 1). Given that the calculations should be accurate to within ±0.21 S (43), both sets of modeled *s*_20,*w*_^0^ values agree well with the experimental values.

## DISCUSSION

The large body of x-ray scattering data permitted a detailed appraisal of the solution structure of human IgG4, and this was supported by complementary neutron scattering and ultracentrifugation experiments. The scattering data enabled atomistic conformational analyses of IgG4 that resulted in three independent determinations of an asymmetric IgG4 solution structure. The combination of the resulting three IgG4 solution structures with (i) a recent docking model for the interaction between human Fc and the crystal structure of the C1q globular head (19, 50) and (ii) crystal structures for the human Fc-FcγR receptor (51, 52) has provided new clarity on the lack of reactivity of human IgG4 with its two major effector ligands.

IgG4 has the lowest IgG serum concentration of the four IgG subclasses at an average level of 0.4 mg/ml (in a range of 0.2–1 mg/ml). The data collection of our IgG4 study utilized a concentration range of 0.4–8.0 mg/ml, which spanned the serum range and above. IgG1 is the most common subclass at 8 mg/ml (in a range of 5–12 mg/ml) followed by 4 mg/ml for IgG2 (in a range of 2–6 mg/ml) and 0.8 mg/ml for IgG3 (in a range of 0.5–0.1 mg/ml) (1). Although distinct immune roles for each of the four IgG subclasses are yet to be elucidated, IgG4 is thought to have anti-inflammatory properties, and the plasma levels of IgG4 rise in response to repeated antigen exposure. IgG4 is unable to activate complement via C1q binding in the complement classical pathway and binds weakly to FcγR receptors (9). IgG4 also has the ability to form half-mers comprising a single pair of light and heavy chains; thus IgG4 is able to undergo Fab-arm switching and become bispecific.

### 

#### 

##### Solution Structure of Wild-type IgG4(Ser^222^)

For IgG4(Ser^222^), our x-ray data collection involved measurement of 192 curves (or 1920 curves if time frames are included) within 2 h (Fig. 3). This high data collection rate permitted the study of its solution structure in different buffers as well as the comparison of the wild-type and mutant forms. Because antibodies are considered to possess flexible hinge structures, the use of three NaCl concentrations will test the potential effects of flexibility on the IgG4 structure. In addition, neutrons permitted the study of IgG4 in heavy water buffer at three temperatures (Fig. 3). All six conditions were replicated using ultracentrifugation (Fig. 1). In contrast, earlier studies reported few scattering and ultracentrifugation runs, which limited the evaluation of potential buffer-dependent IgG4 conformations (45–48).

Despite an expectation that flexible hinge structures would be revealed by scattering, our wild-type IgG4(Ser^222^) data collection indicated no observable change in the *R_g_*, M1/M2, or *s*_20,*w*_^0^ values in concentrations between 2 and 8 mg/ml in six buffers and temperatures (Figs. 3 and 5). This outcome suggests lack of conformational change in its averaged solution structure. However, below 2 mg/ml, both IgG4(Ser^222^) and IgG4(Pro^222^) showed reproducible concentration-dependent changes in the M2 peak that monitors the mean separation between the Fab-Fab and Fab-Fc pairs (Fig. 5). These M2 changes were similar to those reported previously by us for IgG4(Ser^222^) (20). Thus, in the most dilute conditions, the Fab regions underwent 1–2-nm movements relative to the Fc region to affect M2 but not the *R_g_* or *s*_20,*w*_^0^ values. Potential molecular mechanisms for this rearrangement are discussed below.

The scattering modeling fits revealed asymmetric IgG4 molecular structures for the Fab and Fc regions and their connecting hinge in which (i) the IgG4 hinge structures are extended in solution and (ii) the arrangement of the Fab and Fc regions is unaffected by different buffers. IgG4 was modeled starting from the intact IgG1 b12 molecule in which the lower hinge was asymmetrically arranged (37). To avoid bias in our modeling, both asymmetric and symmetric hinge structures were tested (“Experimental Procedures”). All 10 modeling fits showed that its Fab and Fc arrangement was asymmetric. These asymmetric solution structures (Figs. 6–8) are the consequence of its relatively short hinge sequence and the need to accommodate both Fab regions close to the Fc region. Thus the hinge is too short to permit unrestricted conformations of the Fab regions about the Fc region. The IgG4 upper hinge is 7 residues long. Its middle hinge is 5 residues long and contains two interchain disulfide bonds. In the IgG4 models, the α-carbon distances between the Cys^220^-Cys^220^ and Cys^223^-Cys^223^ pairs turned out to be within acceptable limits. There is evidence that the inter- and intrachain disulfide bonds are in equilibrium (13); therefore the hinge disulfide bonds are not necessarily maintained intact. Even if these bonds become separated, IgG4 remains intact due to contacts between the C_H_3 domain pairs as shown by sedimentation velocity (Fig. 1). These asymmetric solution structures resemble crystal structures for intact IgG. Three asymmetric structures include human IgG1 b12 (Protein Data Bank code 1HZH (37)), murine IgG2a 231 (Protein Data Bank code 1IGT (53)), and murine IgG1 61.1.3 (Protein Data Bank code 1IGY (54)). A fourth hinge-deleted human IgG1 Mcg structure, however, showed a symmetric structure (Protein Data Bank code 1MCO (55)). Our results differ from a previous determination of a symmetric structure for wild-type IgG4 based on simple ellipsoid models for the Fab and Fc regions; large differences in that study between the modeled and experimental x-ray curves do not substantiate this symmetric structure (see Fig. 4d of Ref. 45). This is in contrast to our improved curve fits from atomistic modeling based on asymmetric structures (Fig. 7).

After this study was completed, two crystal structures for human IgG4 Fc were published, namely those for a recombinant and a serum-derived Fc fragment (Protein Data Bank codes 4C54 and 4C55 (56). These structures showed conformational variability around Arg^409^ and a unique conformation of the FG loop in the C_H_2 domain when compared with human IgG1 Fc. These two structural features were linked with the Fab-arm exchange behavior and the reduced binding of FcRs to IgG4, respectively. Our homology-modeled Fc structure in the IgG4(Ser^222^) best fit models was compared with the IgG4 Fc crystal structures. The root mean square difference in α-carbon positions when superimposed was low at 0.0937 and 0.0841 nm (Protein Data Bank codes 4C54 and 4C55, respectively). When the Fc region in the scattering best fit model for IgG4(Ser^222^) was replaced with the Fc crystal structures, the resulting *R*-factors of 2.7 (Protein Data Bank code 4C54) and 2.9% (Protein Data Bank code 4C55) were almost unchanged compared with that of 2.8% for the best fit model (Table 1).

##### Comparison of Wild-type and Mutant IgG4

Wild-type and mutant IgG4 differ through the S222P substitution in its hinge. This change to Pro abolishes half-mer formation and presumably results from the reduced flexibility of the hinge (11, 12). Our experimental data showed that the wild-type and mutant forms had similar experimental *R_g_* and *R*_XS_ values (Table 1). Although IgG4(Pro^222^) has a slightly shorter overall length at 15 nm compared with 16 nm for IgG4(Ser^222^), its *s*_20,*w*_^0^ value is slightly smaller at 6.6 S compared with 6.8 S for IgG4(Ser^222^). Both forms showed decreased *s*_20,*w*_^0^ values when the concentration went below 2 mg/ml. Experimentally both forms were indistinguishable within error.

The best fit structures for IgG4(Ser^222^) are predominantly asymmetric, especially at the highest concentrations (Fig. 8*A*). At lower concentrations, both IgG4(Ser^222^) and IgG4(Pro^222^) displayed a combination of asymmetric and symmetric best fit structural models. Although the IgG4(Pro^222^) form is thought to be less flexible (12), the modeling showed little difference between the two forms. The modeling showed that the hinge in both IgG4 forms is mostly extended. This hinge length is measured by the α-carbon positions of the flanking residues Val^212^ and Pro^232^ with a maximum possible length of 7.0 nm. The five IgG4(Ser^222^) hinge lengths decreased slightly with increasing concentration (Fig. 8*A*). In fact, each of the three final best fit structures gave similar hinge lengths of 4.99–5.73 ± 1.08 nm for IgG4(Ser^222^) and 4.81–5.94 ± 1.08 nm for IgG4(Pro^222^) in PBS-137 and 5.58 ± 1.10 nm for IgG4(Ser^222^) in PBS-137 in heavy water.

##### Restricted Interaction of Human IgG4 with C1q

Our full structural models for IgG4 permit the re-evaluation of why C1q is not able to bind to IgG4. Initially C1q had been represented by a sphere of the same volume as its crystal structure so that C1q binding to its Fc binding site could be studied by classic solvent accessibility calculations (20, 57). This simplified approach suggested that the Fab regions in IgG4 obstructed the IgG4 interaction with C1q. Here we performed molecular docking of the Fc and C1q crystal structures to re-evaluate this interaction. This approach had previously shown that the rabbit IgG interaction with C1q was allowed (19, 50).

**FIGURE 9.**
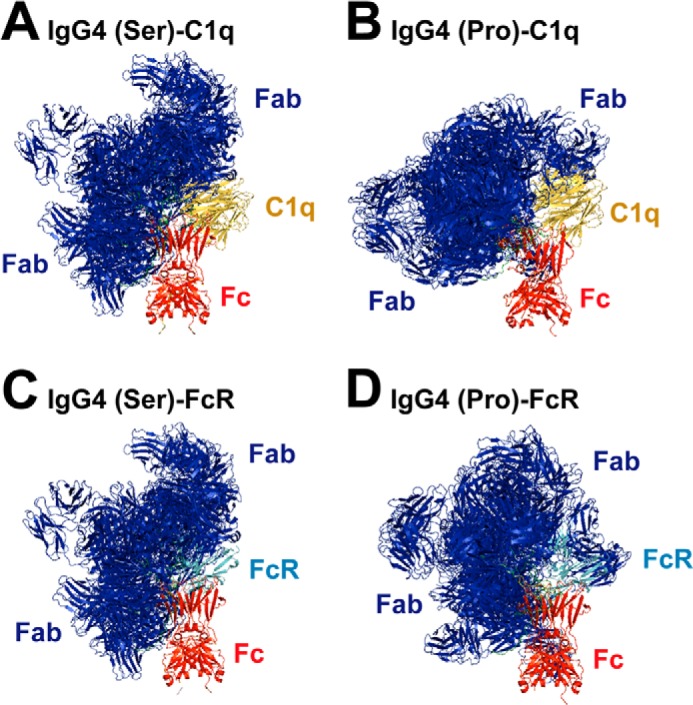
**Superimposition of the best fit IgG4 models with C1q and FcR.** The 10 best fit models are shown superimposed upon their Fc region (*red*) upon which the C1q globular head (*yellow*; Protein Data Bank code 1PK6) (*A* and *B*) or the Fc (*red*)-FcγRIII (*cyan*) complex (Protein Data Bank code 1E4K) (*C* and *D*) are also superimposed. Other Fc-FcR crystal structures (Protein Data Bank codes 1T83 and 1T89) give similar views (not shown). *A*, C1q head with the x-ray models for 5.8 mg/ml IgG4(Ser^222^) in PBS-137. *B*, C1q head with the x-ray models for 2.0 mg/ml IgG4(Pro^222^) in PBS-137. *C*, FcγRIII with the x-ray models for 5.8 mg/ml IgG4(Ser^222^) in PBS-137. *D*, FcγRIII with the x-ray models for 2.0 mg/ml IgG4(Pro^222^) in PBS-137.

The reactivity of complement C1q with the four human IgG subclasses correlates with hinge length in the order IgG3 > IgG1 > IgG2 > IgG4) (1). Given that human IgG4 with the shortest hinge length does not activate complement, this implies that the hinge length is also important for activation. Human IgG residues implicated in C1q binding include Asp^270^, Lys^322^, Pro^329^, and Pro^331^ in the Fc region (58, 59). This C1q binding site occurs at the top of the C_H_2 domain in the Fc region. The Fab regions in both our IgG4(Ser^222^) and IgG4(Pro^222^) models are positioned close to the IgG4 binding site for C1q, potentially hindering C1q binding. Docking studies were performed using a shape complementarity method by the PatchDock server (19). This was based on the 19 C1q and 12 Fc contact residues identified for the IgG1-C1q complex (see Table 2 in Ref. 50). It should be noted that 2 residues were substituted in IgG4 when compared with IgG1 (His^268^ with Gln^268^ and Pro^331^ with Ser^331^, denoted as Gln^262^ and Ser^325^, respectively, in supplemental Fig. S2). In contrast to the rabbit IgG-C1q interaction, our docked IgG4-C1q structures (Fig. 9, *A* and *B*) show that the C1q head is sterically hindered by the Fab regions from interacting with the top of the Fc region in IgG4. In addition, biochemical studies using domain-switched chimeric antibodies showed that the differential complement activation of the subclasses resided in the COOH-terminal C_H_2 domain (60). Mutagenesis studies showed that Ser^331^ in IgG4 was critical for the inability of IgG4 to bind C1q and activate complement. Interestingly, the strength of C1q binding to IgG4 was distinct from its ability to activate complement; thus changes in other parts of IgG4 may be necessary for effective complement activation (61). In conclusion, a combination of steric hindrance from the Fab regions in IgG4, the restricted movement of the IgG4 hinges, and the importance of Ser^331^ for the Fc-C1q interaction appear to account for the lack of binding of C1q to IgG4. The unique conformation of the FG loop in the C_H_2 domains in the crystal structure of IgG4-Fc on which Ser^331^ is located is likely to be relevant too (56).

##### Restricted Interaction of Human IgG4 with FcγR

Many distinct FcγRs are present on immune cells, including monocytes, neutrophils, platelets, and lymphocytes (1), with diverse functions within the immune system. In general, FcγR binding to IgG activates antibody-dependent cell-mediated cytotoxicity events, although individual FcγRs may have activator or inhibitor effects. Differences between the activities of the four IgG subclasses are attributed to their individual affinity for specific FcγRs, hence stimulating a specific and predictable response to certain pathogens (62). The affinities of the four human IgG subclasses for specific FcγRs (FcγRI, FcγRII, and FcγRIII) differ due to different contact residues in the Fc fragment and the FcγRs. Although there is only one high affinity FcγRI receptor, FcγRII and FcγRIII form two families of low affinity receptors together with polymorphic variants as follows: FcγRIIA (His/Arg^131^ allotypes), FcγRIIB/FcγRIIC, FcγRIIIA (Phe/Val^158^ allotypes), and FcγRIIIB (three allelic variants termed NA1 (Arg^36^, Asn^65^, Asp^82^, and Val^106^), NA2 (Ser^36^, Ser^65^, Asn^82^, and Ile^106^), and SH (Ser^36^, Ser^65^, Ala^78^, Asn^82^, and Ile^106^)). The affinity constants (*K_A_*) for binding of the four IgG subclasses with all nine FcγR variants have been established (63). For FcγRI, IgG1 and IgG3 bind most strongly (*K_A_* values of 6.5 and 6.1 × 10^7^ m^−1^, respectively), IgG4 binding is slightly weaker (3.4 × 10^7^ m^−1^), and IgG2 displayed no measureable binding. For the remaining FcγRII and FcγRIII subclasses, IgG4 showed low affinity binding with *K_A_* values near 2 × 10^7^ m^−1^ for FcγRIIA (His/Arg^131^), FcγRIIB/FcγRIIC, and FcγRIIIA (Phe/Val^158^) and no measureable binding to FcγRIIIB (NA1, NA2, and SH allelic variants). In contrast, IgG1 and IgG3 bound to all the FcγRII and FcγRIII subclasses and allelic variants often with higher *K_A_* values ranging between 1.2 × 10^5^ and 9.8 × 10^6^ m^−1^. Conversely, IgG2 showed mostly lower affinities than IgG1, IgG3, and IgG4 for all FcγRs, including no measurable binding for FcγRIIIB (63).

The IgG Fc residues implicated in FcγR binding include Leu^234^-Leu^235^-Gly^236^-Gly^236^-Gly^237^-Pro^238^ of the lower hinge region (64). The Fc glycan interactions with FcγR are important to stabilize the interaction (65, 66). A 1:1 stoichiometry of this interaction has been determined by sedimentation equilibrium (67, 68). The ability of IgG to bind two FcγR molecules may be sterically hindered due to a conformational change upon binding of the first FcγR molecule (69). Crystal structures of the IgG1 Fc-FcγRIIIB complex show that FcγR binds to the top of the Fc region close to the hinge (51, 52). The corresponding IgG4-FcγR interaction was revealed by superimposition of our best fit IgG4(Ser^222^) and IgG4(Pro^222^) solution structures on these crystal structures. This showed that FcγR binding appears to be permitted. Given that the Fab regions in our IgG4 models are close to the top of the Fc region where the FcγRs bind, these Fab regions may offer steric hindrance to binding (Fig. 9, *C* and *D*). However, as IgG4 is able to bind most FcγRs (albeit not as strongly as the IgG1 and IgG3 subclasses), a small movement of the Fab region in our IgG4 models may permit binding to take place, and the strength of binding between Fc and FcγR may be primarily determined by the residues present in the hinge and C_H_2 domains. In this context, IgG4 possesses Phe^234^ in comparison with IgG1 and IgG3 in which this is Leu^234^ and with IgG2, which has a shorter lower hinge region with Val^234^ and Ala^236^ substitutions and the deletion of Leu^235^. IgG4 also has a shorter overall hinge length, which may lead to weaker FcγR binding (70). The higher affinity of IgG4 for FcγRI may be due to an additional extracellular domain in comparison with the two in FcγRII and FcγRIII or structural differences in this additional domain, leading to its greater affinity (71).

##### Stability of Human IgG4

Antibody stability is a major concern in the context of the >$30 billion antibody industry. Stabilities may be compromised during manufacturing, shipping, and storage (72). In the context of bioprocessing, the internal stability of human IgG4 was explored here using different buffers and temperatures by x-ray and neutron scattering. The movement of the M2 peaks below 2 mg/ml confirmed our earlier observations (20). This M2 shift revealed an internal structural rearrangement of the Fab and Fc regions, although here we were not able to model this. In addition, our x-ray and neutron data showed that this rearrangement was stabilized at concentrations higher than 2 mg/ml (Fig. 5). As similar movements of M2 were seen in different NaCl concentrations, this indicated that charge effects within IgG4 or between IgG4 molecules were not responsible for this change. The M2 peak shifts may relate to a diffusion-collision phenomenon (which would be charge-independent) in which more frequent collisions between IgG4 molecules at higher concentrations would lead to a more compact molecular conformation for IgG4. Alternatively the M2 peak shifts may relate to Fab-arm exchange in which the half-mers in IgG4 are more likely to rearrange themselves and dissociate in dilute conditions, leading to conformational instabilities.

To understand better how a diffusion-collision process might lead to conformational change in IgG4, this process can be considered as an equilibrium where collisions between IgG4 molecules at higher concentrations lead to a deformed shape that can relax back to the unperturbed shape in dilute solution. Above 2 mg/ml, the rate of collisions (*k*_coll_) would be greater than the relaxation rate, leading to a deformed IgG4 structure. The upper limit for the relaxation rate is therefore defined by the collision rate constant *k*_coll_ for 2 mg/ml. The collisional frequency *Z* can be calculated using the diffusion coefficient from SEDFIT and the radius of IgG4 from Equation 4.

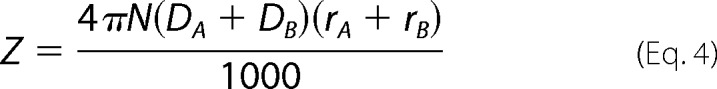
 where *N* is Avogadro's number (6.02214 × 10^−23^), *D* is the diffusion coefficient (4.07 × 10^−11^ m^2^ s^−1^), and *r* is the radius for two IgG4 monomers *A* and *B*. This gives a collision frequency of 6300 m^−1^ s^−1^. The *k*_coll_ value can be calculated from Equation 5.

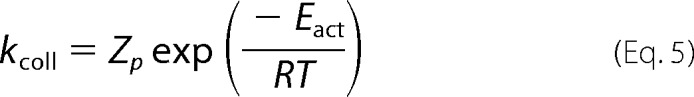
 If the transmission factor *p* is 1 (*i.e.* collisions between molecules always lead to a conformational change) and *E*_act_ is 0, then the equation is simplified to *k*_coll_ = *Z*. At 2 mg/ml (14 μm), the upper limit for the relaxation rate is obtained by multiplying *k*_coll_ by the concentration to give 88 s^−1^. Because the calculation assumes that all collisions lead to a shape change, which is unlikely, the true rate of relaxation will be lower than 88 s^−1^.

Fab-arm exchange through half-mer formation leads to bispecificity in human IgG4 (7). This process leads to the exchange of therapeutic IgG4 molecules with endogenous IgG4 molecules *in vivo* (8). Fab-arm exchange was shown to be a dynamic process (17), and the mechanism of Fab-arm exchange is controlled by local redox conditions (18). Human IgG4 also can exchange with isolated IgG4 Fc fragments, indicating that it is the Fc region that is important for Fab-arm exchange (10). Half-mer was observed for both IgG4(Ser^222^) and IgG4(Pro^222^) by non-reducing SDS-PAGE as a faint band at ∼75 kDa (supplemental Fig. S3). However, these bands have been shown to be artifacts formed under the harsh conditions of SDS-PAGE and depend on heating times and the SDS buffers (73). No half-mer peaks were directly observed in this study by gel filtration or analytical ultracentrifugation (Fig. 1). This indicated that Fab-arm exchange via half-mer formation is slow, but the half-mer life time is short. The low population of half-mer would not be visible by our gel filtration or analytical ultracentrifugation experiments. The movement of the M2 peaks in dilute conditions may predispose the IgG4 molecules to exchange half-mers, perhaps following collisions between two different IgG4 molecules.

Another aspect relevant to antibody bioprocessing relates to IgG4 dimer formation and self-association. Extensive dimer formation may lead to nonspecific antibody aggregation. Indeed, rabbit IgG was observed to form as much as 25% dimer in heavy water buffers, possibly arising through Fab-Fab pairings between the two monomers (19). The low amount of IgG4 dimer observed by sedimentation velocity in six different buffers (Fig. 1) showed that IgG4 does not undergo extensive buffer-dependent dimerization. The dimer peak has an *s*_20,*w*_^0^ value of about 10 S, suggesting that dimers may arise from a compact association of the Fab and Fc regions. However, a concentration-dependent reaction boundary was observed for IgG4(Ser^222^) in heavy water, especially at reduced temperature (Fig. 1*B*). This indicated fast on/fast off association rates between monomer and dimer. Neutron scattering confirmed the temperature-dependent self-association in heavy water (Fig. 3*B*). Greater dimer formation in heavy water is attributed to weaker hydrogen bonding in this solvent, meaning that protein-protein interactions are promoted. In other studies, IgG4 has been shown to associate with the Fc fragments of other IgG classes, probably occurring between their C_H_3 domains (10). The self-association of IgG4 molecules through the C_H_3 domains may be an intermediate before half-mer swapping. IgG4 can also associate with IgG1 following a conformational change of IgG1 that exposes the IgG1 C_H_3 domains (3). We conclude that dimer formation and self-association events may be relevant to the bioprocessing properties of IgG4.

## Footnotes

3 J. T. Eaton and S. J. Perkins, unpublished software.

## Footnotes

2 The abbreviations used are:

FcR: Fc receptor
NeuNAc: *N*-acetylneuraminic acid
*R_g_*: radius of gyration
*R*_XS_: radius of gyration of cross-sectional structure.

## References

[1] Hamilton R. G. (2001) The Human IgG Subclasses, Calbiochem, La Jolla, CA

[2] Aalberse R. C. Schuurman J. van Ree R. (1999) The apparent monovalency of human IgG4 is due to bispecificity. Int. Arch. Allergy Immunol. 118, 187–189 10224373 10.1159/000024062

[3] Rispens T. Ooievaar-De Heer P. Vermeulen E. Schuurman J. van der Neut Kolfschoten M. Aalberse R. C. (2009) Human IgG4 binds to IgG4 and conformationally altered IgG1 via Fc-Fc interactions. J. Immunol. 182, 4275–4281 19299726 10.4049/jimmunol.0804338

[4] Aalberse R. C. Schuurman J. (2002) IgG4 breaking the rules. Immunology 105, 9–19 11849310 10.1046/j.0019-2805.2001.01341.x PMC1782638

[5] Wang X. Das T. K. Singh S. K. Kumar S. (2009) Potential aggregation prone regions in biotherapeutics: a survey of commercial monoclonal antibodies. MAbs 1, 254–267 20065649 10.4161/mabs.1.3.8035 PMC2726584

[6] Aalberse R. C. Stapel S. O. Schuurman J. Rispens T. (2009) Immunoglobulin G4: an odd antibody. Clin. Exp. Allergy. 39, 469–477 19222496 10.1111/j.1365-2222.2009.03207.x

[7] Schuurman J. Van Ree R. Perdok G. J. Van Doorn H. R. Tan K. Y. Aalberse R. C. (1999) Normal human immunoglobulin G4 is bispecific: it has two different antigen-combining sites. Immunology 97, 693–968 10457225 10.1046/j.1365-2567.1999.00845.x PMC2326875

[8] Labrijn A. F. Buijsse A. O. van den Bremer E. T. Verwilligen A. Y. Bleeker W. K. Thorpe S. J. Killestein J. Polman C. H. Aalberse R. C. Schuurman J. van de Winkel J. G. Parren P. W. (2009) Therapeutic IgG4 antibodies engage in Fab-arm exchange with endogenous human IgG4 *in vivo*. Nat. Biotechnol. 27, 767–771 19620983 10.1038/nbt.1553

[9] Clark M. R. (1997) IgG effector mechanisms. Chem. Immunol. 65, 88–110 9018874

[10] Rispens T. den Bleker T. H. Aalberse R. C. (2010) Hybrid IgG4/IgG4 Fc antibodies form upon ‘Fab-arm’ exchange as demonstrated by SDS-PAGE or size-exclusion chromatography. Mol. Immunol. 47, 1592–1594 20299101 10.1016/j.molimm.2010.02.021

[11] Angal S. King D. J. Bodmer M. W. Turner A. Lawson A. D. Roberts G. Pedley B. Adair J. R. (1993) A single amino acid substitution abolishes the heterogeneity of chimeric mouse/human (IgG4) antibody. Mol. Immunol. 30, 105–108 8417368 10.1016/0161-5890(93)90432-b

[12] Bloom J. W. Madanat M. S. Marriott D. Wong T. Chan S. Y. (1997) Intrachain disulphide bond in the core hinge region of human IgG4. Protein Sci. 6, 407–415 9041643 10.1002/pro.5560060217 PMC2143633

[13] Schuurman J. Perdok G. J. Gorter A. D. Aalberse R. C. (2001) The inter-heavy chain disulphide bonds of IgG4 are in equilibrium with intra-heavy chain disulphide bonds. Mol. Immunol. 38, 1–8 11483205 10.1016/s0161-5890(01)00050-5

[14] Labrijn A. F. Rispens T. Meesters J. Rose R. J. den Bleker T. H. Loverix S. van den Bremer E. T. Neijssen J. Vink T. Lasters I. Aalberse R. C. Heck A. J. van de Winkel J. G. Schuurman J. Parren P. W. (2011) Species-specific determinants in the IgG C_H_3 domain enable Fab-arm exchange by affecting the noncovalent C_H_3-C_H_3 interaction strength. J. Immunol. 187, 3238–3246 21841137 10.4049/jimmunol.1003336

[15] Rose R. J. Labrijn A. F. van den Bremer E. T. Loverix S. Lasters I. van Berkel P. H. van de Winkel J. G. Schuurman J. Parren P. W. Heck A. J. (2011) Quantitative analysis of the interaction strength and dynamics of human IgG4 half molecules by native mass spectrometry. Structure 19, 1274–1282 21893287 10.1016/j.str.2011.06.016

[16] Davies A. M. Rispens T. den Bleker T. H. McDonnell J. M. Gould H. J. Aalberse R. C. Sutton B. J. (2013) Crystal structure of the human IgG4 C_H_3 dimer reveals the role of Arg409 in the mechanism of Fab-arm exchange. Mol. Immunol. 54, 1–7 23164605 10.1016/j.molimm.2012.10.029

[17] van der Neut Kolfschoten M. Schuurman J. Losen M. Bleeker W. K. Martínez-Martínez P. Vermeulen E. den Bleker T. H. Wiegman L. Vink T. Aarden L. A. De Baets M. H. van de Winkel J. G. Aalberse R. C. Parren P. W. (2007) Anti-inflammatory activity of human IgG4 antibodies by dynamic Fab-arm exchange. Science 317, 1554–1557 17872445 10.1126/science.1144603

[18] Rispens T. Ooijevaar-de Heer P. Bende O. Aalberse R. C. (2011) Mechanism of immunoglobulin G4 Fab-arm exchange. J. Am. Chem. Soc. 133, 10302–10311 21627172 10.1021/ja203638y

[19] Rayner L. E. Kadkhodayi-Kholghi N. Heenan R. K. Gor J. Dalby P. A. Perkins S. J. (2013) The solution structure of rabbit IgG accounts for its interactions with the Fc receptor and complement C1q and its conformational stability. J. Mol. Biol. 425, 506–523 23178865 10.1016/j.jmb.2012.11.019

[20] Abe Y. Gor J. Bracewell D. G. Perkins S. J. Dalby P. A. (2010) Masking of the Fc region in human IgG4 by constrained x-ray scattering modelling: implications for antibody function and therapy. Biochem. J. 432, 101–111 20722630 10.1042/BJ20100641

[21] Perkins S. J. Bonner A. (2008) Structure determinations of human and chimaeric antibodies by solution scattering and constrained molecular modelling. Biochem. Soc. Trans. 36, 37–42 18208381 10.1042/BST0360037

[22] Brekke O. H. Michaelsen T. E. Sandlie I. (1995) The structural requirements for complement activation by IgG: does it hinge on the hinge? Immunol. Today 16, 85–90 7888072 10.1016/0167-5699(95)80094-8

[23] Lu Y. Harding S. E. Rowe A. J. Davis K. G. Fish B. Varley P. Gee C. Mulot S. (2008) The effect of a point mutation on the stability of IgG4 as monitored by analytical ultracentrifugation. J. Pharm. Sci. 97, 960–969 17722105 10.1002/jps.21016

[24] Owens R. Ball E. Ganesh R. Nesbitt A. Brown D. Gofton C. Stephens S. Chaplin L. Christofidou-Solomidou M. Blake S. Howat D. Buurman W. A. Albelda S. Robinson M. K. (1997) The *in vivo* and in *vitro* characterisation of an engineered human antibody to E-selectin. Immunotechnology 3, 107–116 9237095 10.1016/s1380-2933(97)00066-3

[25] Deisenhofer J. (1981) Crystallographic refinement and atomic models of a human Fc fragment and its complex with fragment B of protein A from *Staphylococcus aureus* at 2.9 and 2.8 Å resolution. Biochemistry 20, 2361–2370 7236608

[26] Boehm M. K. Woof J. M. Kerr M. A. Perkins S. J. (1999) The Fab and Fc fragments of IgA1 exhibit a different arrangement from that in IgG: a study by x-ray and neutron solution scattering and homology modelling. J. Mol. Biol. 286, 1421–1447 10064707 10.1006/jmbi.1998.2556

[27] Perkins S. J. (1986) Protein volumes and hydration effects: the calculation of partial specific volumes, neutron scattering matchpoints and 280 nm absorption coefficients for proteins and glycoproteins from amino acid sequences. Eur. J. Biochem. 157, 169–180 3709531 10.1111/j.1432-1033.1986.tb09653.x

[28] Laue T. M. Shah B. D. Ridgeway T. M. Pelletier S. L. (1992) in Analytical Ultracentrifugation in Biochemistry and Polymer Science (Harding S. E. Rowe A. J. Horton J. C., eds) pp. 90–125, The Royal Society of Chemistry, Cambridge, UK

[29] Schuck P. (1998) Sedimentation analysis of non-interacting and self-associating solutes using numerical solutions to the Lamm equation. Biophys. J. 75, 1503–1512 9726952 10.1016/S0006-3495(98)74069-X PMC1299825

[30] Schuck P. (2000) Size-distribution analysis of macromolecules by sedimentation velocity ultracentrifugation and Lamm equation modelling. Biophys. J. 78, 1606–1619 10692345 10.1016/S0006-3495(00)76713-0 PMC1300758

[31] Narayanan T. Diat O. Bosecke P. (2001) SAXS and USAXS on the high brilliance beamline at the ESRF. Nucl. Instrum. Methods Phys. Res. A 467–468, 1005–1009

[32] Heenan R. K. Rogers S. E. Turner D. Terry A. E. Treadgold J. King S. M. (2011) Small angle neutron scattering using Sans2d. Neutron News 22, 19–21

[33] Glatter O. Kratky O. (1982) Small Angle X-ray Scattering, Academic Press, New York

[34] Pilz I. Kratky O. Licht A. Sela M. (1973) Shape and volume of anti-poly(D-alanyl) antibodies in the presence and absence of tetra-D-alanine as followed by small-angle x-ray scattering. Biochemistry 12, 4998–5005 4796922 10.1021/bi00748a028

[35] Semenyuk A. V. Svergun D. I. (1991) GNOM—a program package for small-angle scattering data-processing. J. Appl. Crystallogr. 24, 537–540 10.1107/S0021889812007662 PMC4233345 25484842

[36] Brady R. L. Hubbard R. E. King D. J. Low D. C. Roberts S. M. Todd R. J. (1991) Crystallization and preliminary x-ray diffraction study of a chimaeric Fab′ fragment of antibody binding tumour cells. J. Mol. Biol. 219, 603–604 2056529 10.1016/0022-2836(91)90656-q

[37] Saphire E. O. Stanfield R. L. Crispin M. D. Parren P. W. Rudd P. M. Dwek R. A. Burton D. R. Wilson I. A. (2002) Contrasting IgG structures reveal extreme asymmetry and flexibility. J. Mol. Biol. 319, 9–18 12051932 10.1016/S0022-2836(02)00244-9

[38] Ashton A. W. Boehm M. K. Gallimore J. R. Pepys M. B. Perkins S. J. (1997) Pentameric and decameric structures in solution of the serum amyloid P component by x-ray and neutron scattering and molecular modelling analyses. J. Mol. Biol. 272, 408–422 9325100 10.1006/jmbi.1997.1271

[39] Perkins S. J. Weiss H. (1983) Low resolution structural studies of mitochondrial ubiquinol:cytochrome c reductase in detergent solutions by neutron scattering. J. Mol. Biol. 168, 847–866 6310128 10.1016/s0022-2836(83)80078-3

[40] Perkins S. J. (2001) X-ray and neutron scattering analyses of hydration shells: a molecular interpretation based on sequence predictions and modelling fits. Biophys. Chem. 93, 129–139 11804721 10.1016/s0301-4622(01)00216-2

[41] Garcia de la Torre J. Navarro S. Lopez Martinez M. C. Diaz F. G. Lopez Cascales J. J. (1994) HYDRO: a computer program for the prediction of hydrodynamic properties of macromolecules. Biophys. J. 67, 530–531 7948671 10.1016/S0006-3495(94)80512-0 PMC1225396

[42] García De La Torre J. Huertas M. L. Carrasco B. (2000) Calculation of hydrodynamic properties of globular proteins from their atomic-level structure. Biophys. J. 78, 719–730 10653785 10.1016/S0006-3495(00)76630-6 PMC1300675

[43] Perkins S. J. Okemefuna A. I. Nan R. Li K. Bonner A. (2009) Constrained solution scattering modelling of human antibodies and complement proteins reveals novel biological insights. J. R. Soc. Interface 6, S679–S696 19605402 10.1098/rsif.2009.0164.focus PMC2843969

[44] Schneidman-Duhovny D. Inbar Y. Nussinov R. Wolfson H. J. (2005) PatchDock and SymmDock: servers for rigid and symmetric docking. Nucleic Acids Res. 33, W363–W367 15980490 10.1093/nar/gki481 PMC1160241

[45] Lu Y. Harding S. E. Michaelsen T. E. Longman E. Davis K. G. Ortega A. Grossmann J. G. Sandlie I. García de la Torre J. (2007) Solution conformation of wild-type and mutant IgG3 and IgG4 immunoglobulins using crystallohydrodynamics: possible implications for complement activation. Biophys. J. 93, 3733–3744 17704171 10.1529/biophysj.107.108993 PMC2084252

[46] Longman E. Kreusel K. Tendler S. B. Fiebrig I. King K. Adair J. O'Shea P. Ortega A. Garcia de la Torre J. Harding S. E. (2003) Estimating domain orientation of two human antibody IgG4 chimeras by crystallohydrodynamics. Eur. Biophys. J. 32, 503–510 12811430 10.1007/s00249-003-0314-y

[47] Byron O. Harding S. Rhind S. (1990) A preliminary investigation of the hydrodynamic properties of two novel monoclonal antibodies. Biochem. Soc. Trans. 18, 1030–1031 2083650 10.1042/bst0181030

[48] Gregory L. Davis K. G. Sheth B. Boyd J. Jefferis R. Nave C. Burton D. R. (1987) The solution conformations of the subclasses of human IgG deduced from sedimentation and small angle x-ray scattering studies. Mol. Immunol. 24, 821–829 3657808 10.1016/0161-5890(87)90184-2

[49] Richardson J. S. Richardson D. C. (1989) in Prediction of Protein Structure and the Principles of Protein Conformation (Fasman G. D., ed) pp. 1–98, Plenum Press, New York

[50] Schneider S. Zacharias M. (2012) Atomic resolution model of the antibody Fc interaction with the complement C1q component. Mol. Immunol. 51, 66–72 22425350 10.1016/j.molimm.2012.02.111

[51] Sondermann P. Huber R. Oosthuizen V. Jacob U. (2000) The 3.2 Å crystal structure of the human IgG1 Fc fragment-FcγRIII complex. Nature 406, 267–273 10917521 10.1038/35018508

[52] Radaev S. Motyka S. Fridman W.-H. Sautes-Fridman C. Sun P. D. (2001) The structure of a human type III Fcγ receptor in complex with Fc. J. Biol. Chem. 276, 16469–16477 11297532 10.1074/jbc.M100350200

[53] Harris L. J. Larson S. B. Hasel K. W. McPherson A. (1997) Refined structure of an intact IgG2a monoclonal antibody. Biochemistry 36, 1581–1597 9048542 10.1021/bi962514+

[54] Harris L. J. Skaletsky E. McPherson A. (1998) Crystallographic structure of an intact IgG1 monoclonal antibody. J. Mol. Biol. 275, 861–872 9480774 10.1006/jmbi.1997.1508

[55] Guddat L. W. Herron J. N. Edmundson A. B. (1993) Three-dimensional structure of a human immunoglobulin with a hinge deletion. Proc. Natl. Acad. Sci. U.S.A. 90, 4271–4275 8483943 10.1073/pnas.90.9.4271 PMC46488

[56] Davies A. M. Rispens T. Ooijevaar-de Heer P. Gould H. J. Jefferis R. Aalberse R. C. Sutton B. J. (2014) Structural determinants of unique properties of human IgG4-Fc. J. Mol. Biol. 426, 630–644 24211234 10.1016/j.jmb.2013.10.039 PMC3905167

[57] Lee B. Richards F. M. (1971) The interpretation of protein structures: estimation of static accessibility. J. Mol. Biol. 55, 379–400 5551392 10.1016/0022-2836(71)90324-x

[58] Thommesen J. E. Michaelsen T. E. Løset G. Å. Sandlie I. Brekke O. H. (2000) Lysine 322 in the human IgG3 C_H_2 domain is crucial for antibody dependent complement activation. Mol. Immunol. 37, 995–1004 11395138 10.1016/s0161-5890(01)00010-4

[59] Idusogie E. E. Presta L. G. Gazzano-Santoro H. Totpal K. Wong P. Y. Ultsch M. Meng Y. G. Mulkerrin M. G. (2000) Mapping of the C1q binding site on Rituxan, a chimeric antibody with human IgG1 Fc. J. Immunol. 164, 4178–4184 10754313 10.4049/jimmunol.164.8.4178

[60] Tao M. H. Canfield S. M. Morrison S. L. (1991) The differential ability of human IgG1 and IgG4 to activate complement is determined by the COOH-terminal sequence of the C_H_2 domain. J. Exp. Med. 173, 1025–1028 2007852 10.1084/jem.173.4.1025 PMC2190803

[61] Tao M. H. Smith R. I. Morrison S. L. (1993) Structural features of human immunoglobulin G that determine isotype-specific differences in complement activation. J. Exp. Med. 178, 661–667 8340761 10.1084/jem.178.2.661 PMC2191116

[62] Nimmerjahn F. Ravetch J. V. (2005) Divergent immunoglobulin G subclass activity through selective Fc receptor binding. Science 310, 1510–1512 16322460 10.1126/science.1118948

[63] Bruhns P. Iannascoli B. England P. Mancardi D. A. Fernandez N. Jorieux S. Daëron M. (2009) Specificity and affinity of human Fcγ receptors and their polymorphic variants for human IgG subclasses. Blood 113, 3716–3725 19018092 10.1182/blood-2008-09-179754

[64] Tamm A. Schmidt R. E. (1997) IgG binding sites of human Fcγ receptors. Int. Rev. Immunol. 16, 57–85 9651786 10.3109/08830189709045703

[65] Krapp S. Mimura Y. Jefferis R. Huber R. Sondermann P. (2003) Structural analysis of human IgG-Fc glycoforms reveals a correlation between glycosylation and structural integrity. J. Mol. Biol. 325, 979–989 12527303 10.1016/s0022-2836(02)01250-0

[66] Nimmerjahn F. Ravetch J. V. (2008) Fcγ receptors as regulators of immune responses. Nat. Rev. Immunol. 8, 34–47 18064051 10.1038/nri2206

[67] Ghirlando R. Keown M. B. Mackay G. A. Lewis M. S. Unkeless J. C. Gould H. J. (1995) Stoichiometry and thermodynamics of the interaction between the Fc fragments of human IgG1 and its low-affinity receptor FcγRIII. Biochemistry 34, 13320–13327 7577916 10.1021/bi00041a007

[68] Kato K. Fridman W. H. Arata Y. Sautès-Fridman C. (2000) A conformational change in the Fc precludes the binding of two Fcy receptor molecules to one IgG. Immunol. Today 21, 310–312 10871868 10.1016/s0167-5699(00)01666-2

[69] Kato K. Sautès-Fridman C. Yamada W. Kobayashi K. Uchiyama S. Kim H. Enokizono J. Galinha A. Kobayashi Y. Fridman W. H. Arata Y. Shimada I. (2000) Structural basis of the interaction between IgG and Fcγ receptors. J. Mol. Biol. 295, 213–224 10623521 10.1006/jmbi.1999.3351

[70] Radaev S. Sun P. (2002) Recognition of immunoglobulins by Fcγ receptors. Mol. Immunol. 38, 1073–1083 11955599 10.1016/s0161-5890(02)00036-6

[71] Lu J. Ellsworth J. L. Hamacher N. Oak S. W. Sun P. D. (2011) Crystal structure of Fcγ receptor I and its implication in high affinity γ-immunoglobulin binding. J. Biol. Chem. 286, 40608–40613 21965667 10.1074/jbc.M111.257550 PMC3220521

[72] Wang W. Singh S. Zeng D. L. King K. Nema S. (2007) Antibody structure, instability and formulation. J. Pharm. Sci. 96, 1–26 16998873 10.1002/jps.20727

[73] Taylor F. R. Prentice H. L. Garber E. A. Fajardo H. A. Vasilyeva E. Blake Pepinsky R. (2006) Suppression of sodium dodecyl sulfate-polyacrylamide gel electrophoresis sample preparation artifacts for analysis of IgG4 half-antibody. Anal. Biochem. 353, 204–208 16564018 10.1016/j.ab.2006.02.022

